# Global, regional, and national disease burden of paralytic ileus and intestinal obstruction among individuals younger than 20 years, 1990 to 2021: Findings from the Global Burden of Disease Study 2021

**DOI:** 10.1097/MD.0000000000049668

**Published:** 2026-07-10

**Authors:** Xia Yan, Yaohui Dong, Fangbing Li, He Yang, Meilin Zhang, Huina Yu

**Affiliations:** a Department of Pediatrics, Jingzhou Hospital Affiliated to Yangtze University, Jingzhou, Hubei, China; b Department of Cardiovascular Medicine, Jingzhou Hospital Affiliated to Yangtze University, Jingzhou, Hubei, China; c Department of Infectious Diseases, Tianjin Medical University General Hospital, Tianjin, China.

**Keywords:** Bayesian forecasting, Global Burden of Disease, health inequities, paralytic ileus and intestinal obstruction

## Abstract

Using Global Burden of Disease Study 2021 data, this study assessed the global, regional, and national burden of paralytic ileus and intestinal obstruction among individuals younger than 20 years from 1990 to 2021 and explored its temporal patterns, inequalities, drivers, and future trends. We extracted global, regional, and national estimates of incidence, mortality, prevalence, and disability-adjusted life years (DALYs), together with age-standardized rates (ASR). Analytical methods included Joinpoint regression, age–period–cohort analysis, inequality and frontier analysis, decomposition analysis, and Bayesian age–period–cohort modeling to project burden to 2050. From 1990 to 2021, global ASR for incidence, mortality, prevalence, and DALYs declined, whereas absolute case numbers increased due to population growth. The burden was highest among children under 5 years and in lower socio-demographic index (SDI) regions. While inequalities in incidence and prevalence were relatively small, mortality and DALYs were disproportionately concentrated in lower-SDI regions, indicating pro-poor inequality. Frontier analysis revealed efficiency gaps in incidence and prevalence management in high-SDI areas, whereas several low-SDI countries approached their potential minimum for mortality and DALYs. Decomposition analysis attributed declining mortality and DALYs to epidemiological improvement and rising case numbers to population growth. All ASRs are projected to decline or stabilize by 2050. While fatal outcomes have improved, rising case numbers and persistent inequalities define the burden. Mortality reduction is driven by care improvements, while demographic growth fuels case increases. Public health strategies must be targeted: high-SDI regions should enhance prevention, low-SDI regions must strengthen acute care, and all efforts should prioritize children under 5.

## 1. Introduction

Paralytic ileus and intestinal obstruction (PI&IO) are important causes of acute abdominal emergencies in children and adolescents and remain associated with substantial morbidity, mortality, and healthcare burden worldwide.^[1]^ In pediatric populations, PI&IO may arise from a wide range of congenital, inflammatory, infectious, postoperative, and functional conditions, and delayed diagnosis or inadequate treatment may rapidly result in bowel ischemia, perforation, sepsis, and death. Because children, especially younger children, often present with nonspecific symptoms and have limited physiological reserve, timely recognition and access to emergency surgical care are particularly important for improving outcomes.^[2]^ Therefore, PI&IO represents not only a clinical challenge but also a relevant public health issue, particularly in settings with constrained healthcare resources and limited surgical capacity.

The burden of pediatric surgical conditions is unevenly distributed across regions and is closely associated with socioeconomic development, health-system capacity, and access to essential surgical services. According to estimates from the Global Burden of Disease (GBD) Study 2021, PI&IO accounted for a considerable number of incident cases, deaths, and disability-adjusted life years (DALYs) among individuals younger than 20 years, with the greatest burden observed in children under 5 years of age.^[3–5]^ Although age-standardized rates have generally declined over time, the absolute number of affected individuals has continued to increase in many settings, partly because of population growth and persistent inequalities in healthcare delivery. These patterns suggest that the burden of PI&IO is shaped not only by disease occurrence itself, but also by inequities in timely diagnosis, referral, and treatment.^[6,7]^

Recently, Zhao et al assessed the global, regional, and national burden of PI&IO using GBD 2021 data from 1990 to 2021, providing important estimates of incident cases and years of life lost in the overall population.^[8]^ However, evidence focusing specifically on children and adolescents remains limited. Although the GBD framework provides a valuable platform for evaluating long-term epidemiological patterns across countries, regions, age groups, and levels of development using standardized comparative methods, previous studies on PI&IO and related pediatric surgical conditions have often remained largely descriptive, with limited investigation into mortality, prevalence, DALYs, temporal trend heterogeneity, age–period–cohort effects, socioeconomic inequality, the relative contributions of demographic and epidemiological factors to changing burden, health-system performance gaps, and future trends in this vulnerable population.^[9–12]^

Therefore, using data from the GBD 2021 study, the present study aimed to systematically assess the global, regional, and national burden of PI&IO among individuals younger than 20 years from 1990 to 2021. Specifically, we examined incidence, mortality, prevalence, and DALYs; evaluated temporal trends using Joinpoint regression and age–period–cohort analysis; quantified the contributions of population growth, population aging, and epidemiological change through decomposition analysis; assessed health inequalities and performance gaps across socio-demographic index (SDI) levels using inequality and frontier analyses; and projected future burden trends to 2050 using a Bayesian age–period–cohort model.^[13–15]^ By integrating these approaches, this study aims to provide a more comprehensive understanding of the burden of PI&IO and to inform targeted prevention, improved emergency surgical care, and equity-oriented health policy for children and adolescents worldwide.^[16]^

## 2. Methods

### 2.1. Data sources

This retrospective observational study was based on a secondary analysis of publicly available data from the GBD 2021. The study was reported with reference to the Strengthening the Reporting of Observational Studies in Epidemiology Statement.^[17]^ GBD 2021, developed by the Institute for Health Metrics and Evaluation, provides comprehensive estimates for 369 diseases and 88 risk factors across 204 countries and territories from 1990 to 2021. Data were obtained from the Global Health Data Exchange platform (http://ghdx.healthdata.org/gbd-results-tool) and included incidence, mortality, prevalence, DALYs, age-standardized rates (ASR), and SDI for PI&IO. To assess temporal trends, annual percentage change (APC) and estimated annual percentage change (EAPC) were calculated for each indicator. To improve the transparency of the study design and analytical workflow, a flowchart summarizing the data source, study population, burden indicators, and main analytical procedures is provided in Figure S1, Supplemental Digital Content 1.

### 2.2. Analysis of worldwide and regional burden

An assessment of the worldwide and regional disease burden was conducted to evaluate the spatial patterns and geographical inequalities associated with PI&IO. All statistical analyses and visualizations were performed using R software (version 4.4.1; R Foundation for Statistical Computing, Vienna, Austria). The classification of 21 geographical regions followed the framework established by the GBD study.^[18]^

### 2.3. Analysis based on the socio-demographic index (SDI)

The association between the SDI and the burden of PI&IO was examined by deriving disease rates specific to SDI levels.^[19]^ Countries and territories were categorized into 5 SDI quintiles (low, low-middle, middle, high-middle, and high) to analyze the association between SDI and the burden of PI&IO. The strength (*r* coefficients) and significance (*P*-values) of the correlation between ASR and SDI were assessed using Spearman rank analysis. All data processing and visualization were performed in R using the ‘dplyr’ and ‘ggplot2’ packages.

### 2.4. Analysis of temporal trends

To evaluate temporal variations in incidence, mortality, prevalence, and DALYs, a segmented regression model was employed using the ‘segmented’ and ‘broom’ packages in R. The statistical significance of the identified trends was determined by calculating the APC and average annual percentage change (AAPC), each presented with their corresponding 95% uncertainty intervals (UI).^[20,21]^

### 2.5. Age–period–cohort analysis

We performed an age–period–cohort analysis using the R package ‘apc’ to assess the effects of age, period, and birth cohort on PI&IO burden. The net drift (overall temporal trend) and local drift (age-specific trends) represent the APC in age-adjusted and age-specific rates, respectively.^[22]^ The age effect refers to burden variations by age group, the period effect to differences across all ages at different times, and the cohort effect to changes within the same birth cohort over time.

### 2.6. Decomposition analysis

A decomposition analysis was conducted to quantify the contributions of population aging, population growth, and shifts in epidemiological rates to the numerical changes in the burden of PI&IO between 1990 and 2021. This method elucidates how demographic dynamics and changes in disease risk have jointly influenced the observed temporal trends.^[23]^

### 2.7. Analysis of health inequalities

To assess absolute and relative disparities in PI&IO burden, we computed the slope index of inequality (SII) and concentration index (CII).^[24]^ The SII was derived from a robust regression model of age-standardized incidence and mortality rates (ASIR and ASMR) against SDI, using the midpoint of the cumulative population distribution ranked by SDI. The CII was calculated by plotting the cumulative proportion of disease burden against the cumulative population distribution across SDI strata and determining the area under the concentration curve.

### 2.8. Frontier analysis

Frontier analysis was used to estimate the minimum feasible ASIR and ASMR achievable for each region at its current SDI level.^[25]^ These frontier values benchmark optimal performance, allowing assessment of relative health outcome efficiency. The non-linear association between SDI and ASIR/ASMR was modeled using locally weighted regression with multiple smoothing spans (0.3, 0.4, and 0.5) to generate smoothed frontier lines. This quantifies each region’s current performance gap relative to its potential minimum burden, indicating the scope for future improvement.

### 2.9. Forecasting with the Bayesian age–period–cohort (BAPC) Model

To project future trends in the burden of PI&IO, a BAPC model was applied. Utilizing the ‘BAPC’ and ‘INLA’ packages in R, this model forecasts the future numbers and ASRs for incidence, mortality, prevalence, and DALYs. By integrating the effects of age, time period, and birth cohort, it enables the projection of the anticipated disease burden landscape for PI&IO.^[9,26]^

### 2.10. Statistical analysis

All statistical computations and graphical representations were performed with R software (version 4.4.1). Descriptive summaries were computed for primary variables. Metrics sourced directly from the GBD 2021 study: including case numbers, ASR, and percentage change: are reported as means accompanied by 95% UI. Results derived from our analyses, such as the EAPC and projections from the BAPC model, are presented as means with 95% confidence intervals (CI). In trend analyses, statistical significance was defined as a *P*-value of <.05.^[27,28]^

## 3. Results

### 3.1. Global burden of PI&IO from 1990 to 2021

**Figure 1.**
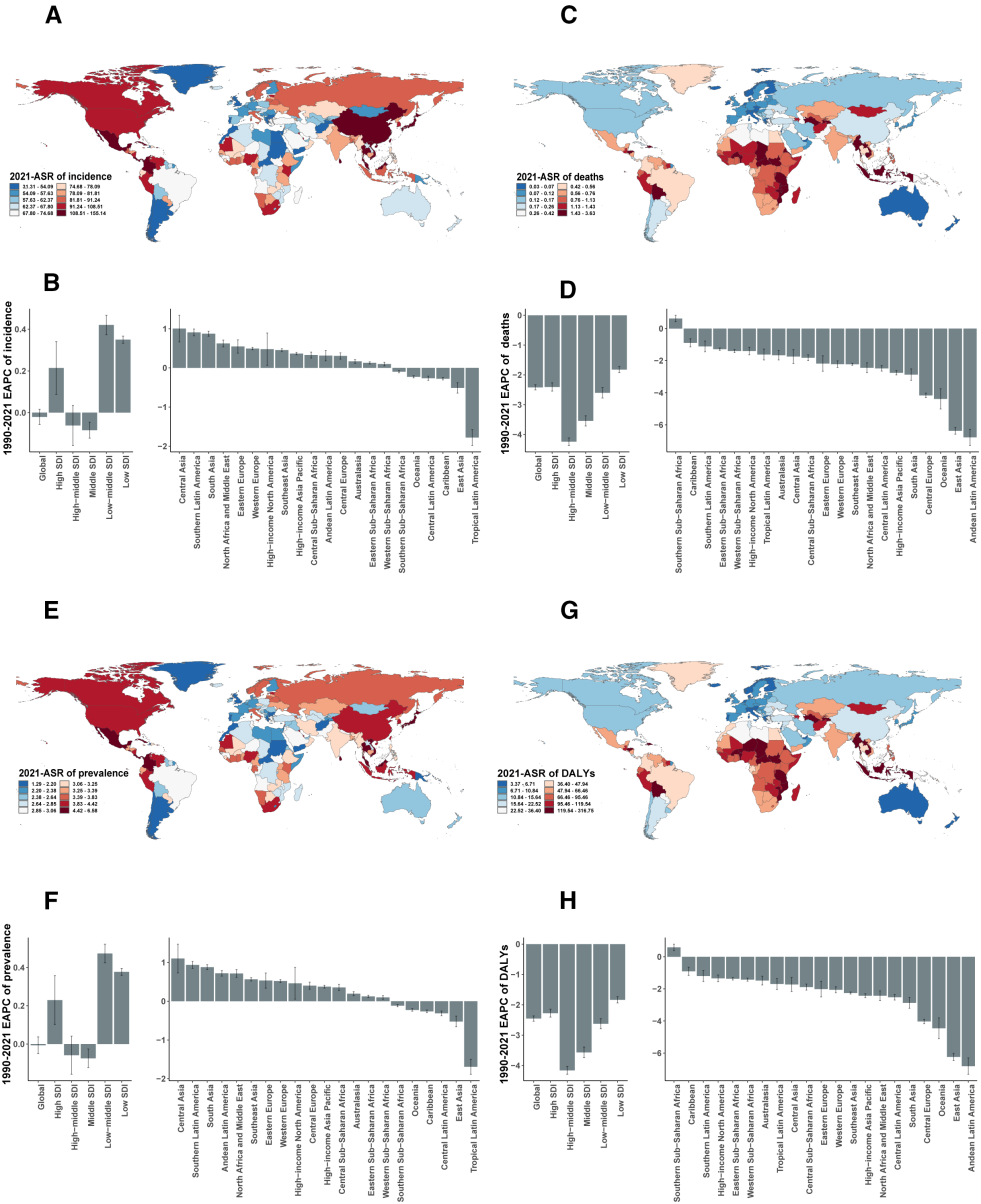
Global disease burden of PI&IO in 2021. Geographic heat map exhibiting ASR of incidence (A) deaths (C) prevalence (E) and DALYs (G) for PI&IO in 2021. Bar chart exhibiting global and regional EAPC of incidence (B) deaths (D), prevalence (F), and DALYs (H) for PI&IO from 1990 to 2021. ASR = age-standardized rate, DALYs = disability-adjusted life years, EAPC = estimated annual percentage change, PI&IO = paralytic ileus and intestinal obstruction.

Globally, the burden of PI&IO among individuals younger than 20 years showed divergent trends between absolute counts and age-standardized rates from 1990 to 2021. Incident cases increased from 1,953,964 in 1990 to 2,210,702 in 2021, whereas the ASIR declined slightly from 86.51 to 83.87 per 100,000, with an EAPC of −0.02. A similar pattern was observed for prevalence: the number of prevalent cases increased from 80,058 to 90,346, while the age-standardized prevalence rate decreased modestly from 3.54 to 3.43 per 100,000 (EAPC: −0.01). In contrast, severe outcomes improved substantially over the same period. Deaths decreased by 48.2%, from 36,190 to 18,728, and the ASMR fell by 55.66%, from 1.60 to 0.71 per 100,000 (EAPC: −2.42). Likewise, DALYs declined from 3161,663 to 1613,343, and the age-standardized DALY rate decreased from 139.98 to 61.21 per 100,000, corresponding to a 56.28% reduction (EAPC: −2.45). Overall, these findings indicate that although the fatal and disabling burden of PI&IO has markedly improved, the absolute number of affected children and adolescents has continued to rise (Tables 1–4, Fig. 1).

**Table 1:** The incidence cases and age-standardized incidence rate of paralytic ileus and intestinal obstruction in 1990 and 2021, and its temporal trends from 1990 to 2021.

Characteristics	1990		2021		1990–2021	1990–2021
	Incidence cases no. ×10^5^ (95% UI)	ASR per 100,000 no. (95% UI)	Incidence cases no. ×10^5^ (95% UI)	ASR per 100,000 no. (95% UI)	Percentage change in age-standardized rates	EAPCs of ASR
Global	1,953,964 (1,832,296–2,073,014)	86.51 (81.13–91.78)	2,210,702 (2,091,026–2,343,155)	83.87 (79.33–88.90)	−3.05 (−4.79 to −1.19)	−0.02 (−0.06 to 0.02)
High SDl	219,678 (204,196–234,605)	87.41 (81.25–93.35)	208,367 (196,532–221,448)	89.53 (84.45–95.16)	2.43 (0.49 to 4.64)	0.21 (0.09 to 0.34)
High-middle SDl	375,807 (348,432–403,993)	101.52 (94.13–109.14)	286,433 (269,761–305,460)	94.42 (88.92–100.69)	−7.00 (−10.04 to −3.44)	−0.06 (−0.16 to 0.03)
Middle SDl	776,703 (727,294–825,827)	101.59 (95.13–108.01)	716,832 (677,739–759,788)	95.68 (90.46–101.41)	−5.82 (−8.05 to −3.57)	−0.08 (−0.12 to −0.05)
Low-middle SDl	400,376 (378,847–421,111)	67.74 (64.10–71.25)	579,877 (547,340–613,800)	75.86 (71.61–80.30)	11.98 (10.27 to 13.55)	0.42 (0.37 to 0.47)
Low SDI	180,079 (170,905–189,319)	64.41 (61.13–67.72)	417,812 (394,070–440,982)	71.52 (67.45–75.48)	11.03 (9.50 to 12.50)	0.35 (0.33 to 0.37)
Andean Latin America	14,856 (14,112–15,550)	78.37 (74.44–82.03)	19,959 (18,889–21,053)	84.31 (79.79–88.93)	7.58 (3.57 to 11.37)	0.31 (0.18 to 0.44)
Australasia	3813 (3554–4102)	60.79 (56.66–65.40)	4791 (4446–5120)	63.53 (58.96–67.89)	4.51 (0.33 to 9.31)	0.17 (0.12 to 0.21)
Caribbean	12,104 (11,471–12,795)	80.17 (75.97–84.74)	11,231 (10,670–11,862)	73.59 (69.91–77.72)	−8.21 (−10.17 to −6.08)	−0.28 (−0.31 to −0.25)
Central Asia	16,963 (16,137–17,769)	53.71 (51.10–56.27)	21,422 (20,333–22,545)	61.87 (58.72–65.11)	15.18 (12.84 to 17.69)	1.00 (0.66 to 1.34)
Central Europe	23,084 (21,696–24,507)	58.78 (55.25–62.41)	14,576 (13,814–15,417)	61.87 (58.64–65.45)	5.26 (2.85 to 8.12)	0.31 (0.22 to 0.39)
Central Latin America	103,181 (96,574–108,865)	124.87 (116.87–131.75)	95,747 (90,529–100,816)	112.27 (106.15–118.21)	−10.09 (−11.84 to −7.91)	−0.26 (−0.32 to −0.21)
Central Sub-Saharan Africa	18,927 (17,860–20,106)	61.08 (57.63–64.88)	49,921 (46,575–52,968)	67.86 (63.31–72.01)	11.11 (7.41 to 15.42)	0.33 (0.25 to 0.41)
East Asia	620,399 (568,184–670,491)	134.83 (123.48–145.71)	375,007 (352,148–400,642)	108.71 (102.09–116.14)	−19.37 (−22.87 to −15.31)	−0.51 (−0.64 to −0.38)
Eastern Europe	55,480 (51,526–59,300)	82.47 (76.59–88.14)	38,927 (36,456–41,532)	84.33 (78.98–89.98)	2.26 (−0.13 to 5.19)	0.55 (0.38 to 0.72)
Eastern Sub-Saharan Africa	80,678 (75,960–84,901)	72.75 (68.50–76.56)	174,037 (164,293–183,944)	76.47 (72.19–80.82)	5.11 (3.23 to 6.92)	0.13 (0.10 to 0.16)
High-income Asia Pacifc	58,652 (54,186–63,703)	116.54 (107.67–126.58)	39,563 (36,898–42,424)	128.50 (119.84–137.79)	10.26 (7.50 to 12.95)	0.37 (0.34 to 0.40)
High-income North America	76,318 (71,239–81,520)	93.38 (87.16–99.74)	88,359 (83,381–93,919)	98.66 (93.10–104.87)	5.66 (3.18 to 8.70)	0.48 (0.07 to 0.89)
North Africa and Middle East	87,548 (82,237–92,970)	49.53 (46.52–52.59)	133,453 (125,477–141,967)	56.43 (53.06–60.03)	13.94 (11.20 to 16.46)	0.63 (0.54 to 0.71)
Oceania	1982 (1860–2114)	58.87 (55.26–62.80)	3583 (3352–3808)	56.11 (52.48–59.63)	−4.70 (−7.91 to −1.19)	−0.23 (−0.25 to −0.20)
South Asia	342,875 (323,275–36,2487)	63.21 (59.60–66.83)	551,065 (518,886–586,545)	80.62 (75.92–85.82)	27.55 (25.33 to 29.88)	0.87 (0.81 to 0.94)
Southeast Asia	182,765 (171,728–192,713)	83.11 (78.09–87.64)	215,214 (202,799–227,372)	93.87 (88.45–99.17)	12.94 (11.27 to 14.81)	0.46 (0.42 to 0.49)
Southern Latin America	6111 (5703–6590)	31.53 (29.43–34.00)	8121 (7569–8714)	41.63 (38.80–44.66)	32.00 (26.84 to 37.51)	0.91 (0.82 to 0.99)
Southern Sub-Saharan Africa	25,807 (24,174–27,443)	97.53 (91.36–103.71)	29,420 (27,498–31,375)	94.10 (87.95–100.35)	−3.52 (−5.55 to −1.35)	−0.10 (−0.12 to −0.07)
Tropical Latin America	80,772 (76,310–85,490)	116.62 (110.18–123.43)	49,078 (46,079–52,281)	73.70 (69.20–78.52)	−36.80 (−38.65 to −34.90)	−1.78 (−1.98 to −1.57)
Western Europe	50,363 (46,909–53,909)	51.21 (47.70–54.82)	53,935 (50,676–57,685)	58.81 (55.26–62.90)	14.84 (12.23 to 17.62)	0.49 (0.46 to 0.52)
Western Sub-Saharan Africa	91,286 (86,330–96,290)	84.92 (80.31–89.58)	233,293 (220,422–246,069)	86.86 (82.07–91.62)	2.29 (0.57 to 3.63)	0.10 (0.06 to 0.14)

ASR = age-standardized rate, EAPC = estimated annual percentage change, UI = uncertainty interval.

**Table 2:** The deaths cases and age-standardized deaths rate of paralytic ileus and intestinal obstruction in 1990 and 2021, and its temporal trends from 1990 to 2021.

Characteristics	1990		2021		1990–2021	1990–2021
	Deaths cases no. ×10^5^ (95% UI)	ASR per 100,000 no. (95% UI)	Deaths cases no. ×10^5^ (95% UI)	ASR per 100,000 no. (95% UI)	Percentage change in age-standardized rates	EAPCs of ASR
Global	36,190 (30,419–42,586)	1.60 (1.35–1.89)	18,728 (14,512–23,209)	0.71 (0.55–0.88)	−55.66 (−66.51 to −38.26)	−2.42 (−2.50 to −2.33)
High SDl	718 (676–770)	0.29 (0.27–0.31)	281 (257–304)	0.12 (0.11–0.13)	−57.78 (−61.54 to −53.91)	−2.41 (−2.54 to −2.27)
High-middle SDl	3401 (2958–3941)	0.92 (0.80–1.06)	674 (563–785)	0.22 (0.19–0.26)	−75.83 (−80.71 to −70.27)	−4.24 (−4.37 to −4.11)
Middle SDl	14,868 (12,627–17,171)	1.94 (1.65–2.25)	4423 (3413–5543)	0.59 (0.46–0.74)	−69.64 (−76.58 to −58.05)	−3.54 (−3.71 to −3.37)
Low-middle SDl	11,080 (8847–13,723)	1.87 (1.50–2.32)	6231 (4816–7775)	0.82 (0.63–1.02)	−56.52 (−68.18 to −33.63)	−2.60 (−2.78 to −2.43)
Low SDI	6094 (4619–7760)	2.18 (1.65–2.78)	7101 (5215–9264)	1.22 (0.89–1.59)	−44.24 (−60.19 to −17.56)	−1.82 (−1.92 to −1.72)
Andean Latin America	1827 (1347–2325)	9.64 (7.11–12.27)	296 (226–388)	1.25 (0.95–1.64)	−87.01 (−91.02 to −80.41)	−6.78 (−7.28 to −6.28)
Australasia	8 (7–8)	0.12 (0.12–0.13)	4 (4–5)	0.05 (0.05–0.06)	−56.14 (−62.60 to −49.02)	−1.66 (−1.95 to −1.37)
Caribbean	301 (205–447)	1.99 (1.36–2.96)	225 (147–326)	1.47 (0.97–2.13)	−26.12 (−49.78 to 11.98)	−0.89 (−1.14 to −0.63)
Central Asia	812 (708–915)	2.57 (2.24–2.90)	379 (296–494)	1.09 (0.86–1.43)	−57.45 (−67.68 to −45.41)	−1.74 (−2.18 to −1.31)
Central Europe	224 (208–241)	0.57 (0.53–0.61)	31 (26–36)	0.13 (0.11–0.15)	−77.17 (−80.94 to −72.12)	−4.17 (−4.31 to −4.02)
Central Latin America	1596 (1443–1750)	1.93 (1.75–2.12)	680 (519–899)	0.80 (0.61–1.05)	−58.74 (−68.33 to −45.49)	−2.48 (−2.65 to −2.31)
Central Sub-Saharan Africa	609 (418–870)	1.96 (1.35–2.81)	733 (420–1376)	1.00 (0.57–1.87)	−49.25 (−71.48 to −11.60)	−1.82 (−2.00 to −1.64)
East Asia	8706 (7201–10,549)	1.89 (1.57–2.29)	852 (666–1081)	0.25 (0.19–0.31)	−86.95 (−90.33 to −82.58)	−6.37 (−6.58 to −6.16)
Eastern Europe	248 (232–266)	0.37 (0.35–0.40)	65 (60–70)	0.14 (0.13–0.15)	−61.75 (−65.35 to −57.77)	−2.18 (−2.66 to −1.70)
Eastern Sub-Saharan Africa	2260 (1658–3000)	2.04 (1.49–2.71)	2962 (2153–3888)	1.30 (0.95–1.71)	−36.13 (−59.14 to 9.01)	−1.29 (−1.36 to −1.21)
High-income Asia Pacifc	113 (100–128)	0.22 (0.20–0.25)	27 (25–29)	0.09 (0.08–0.09)	−61.14 (−66.80 to −55.40)	−2.76 (−2.89 to −2.63)
High-income North America	229 (222–236)	0.28 (0.27–0.29)	149 (136–161)	0.17 (0.15–0.18)	−40.71 (−45.80 to −34.72)	−1.40 (−1.63 to −1.17)
North Africa and Middle East	2023 (1530–2533)	1.14 (0.87–1.43)	1034 (812–1276)	0.44 (0.34–0.54)	−61.80 (−72.58 to −45.43)	−2.45 (−2.75 to −2.14)
Oceania	36 (22–51)	1.06 (0.65–1.51)	21 (14–29)	0.32 (0.22–0.46)	−69.36 (−83.00 to −43.82)	−4.38 (−5.01 to −3.75)
South Asia	7024 (5538–8581)	1.29 (1.02–1.58)	3623 (2613–4776)	0.53 (0.38–0.70)	−59.07 (−72.19 to −36.87)	−2.87 (−3.22 to −2.51)
Southeast Asia	6864 (4545–9440)	3.12 (2.07–4.29)	3592 (2580–4536)	1.57 (1.13–1.98)	−49.80 (−68.06 to −11.67)	−2.23 (−2.29 to −2.16)
Southern Latin America	77 (70–84)	0.40 (0.36–0.43)	43 (38–49)	0.22 (0.19–0.25)	−43.82 (−51.84 to −34.01)	−1.11 (−1.45 to −0.78)
Southern Sub-Saharan Africa	193 (163–226)	0.73 (0.62–0.85)	248 (198–303)	0.79 (0.63–0.97)	8.81 (−15.65 to 37.03)	0.62 (0.42 to 0.82)
Tropical Latin America	647 (562–725)	0.93 (0.81–1.05)	314 (256–371)	0.47 (0.38–0.56)	−49.45 (−60.55 to −36.93)	−1.62 (−1.96 to −1.28)
Western Europe	165 (160–170)	0.17 (0.16–0.17)	69 (63–75)	0.08 (0.07–0.08)	−55.18 (−59.27 to −51.20)	−2.22 (−2.42 to −2.01)
Western Sub-Saharan Africa	2229 (1592–3198)	2.07 (1.48–2.97)	3381 (2345–5054)	1.26 (0.87–1.88)	−39.29 (−56.16 to −15.49)	−1.40 (−1.49 to −1.30)

ASR = age-standardized rate, EAPC = estimated annual percentage change, UI = uncertainty interval.

**Table 3:** The prevalence cases and age-standardized prevalence rate of paralytic ileus and intestinal obstruction in 1990 and 2021, and its temporal trends from 1990 to 2021.

Characteristics	1990		2021		1990–2021	1990–2021
	Prevalence cases no. ×10^5^ (95% UI)	ASR per 100,000 no. (95% UI)	Prevalence cases no. ×10^5^ (95% UI)	ASR per 100,000 no. (95% UI)	Percentage change in age-standardized rates	EAPCs of ASR
Global	80,058 (75,361–84,561)	3.54 (3.34–3.74)	90,346 (85,653–95,381)	3.43 (3.25–3.62)	−3.30 (−5.00 to −1.57)	−0.01 (−0.05 to 0.04)
High SDl	8874 (8296–9457)	3.53 (3.30–3.76)	8415 (7979–8895)	3.62 (3.43–3.82)	2.41 (0.56 to 4.47)	0.23 (0.10 to 0.36)
High-middle SDl	15,665 (14,577–16,742)	4.23 (3.94–4.52)	11,739 (11,086–12,467)	3.87 (3.65–4.11)	−8.56 (−11.48 to −5.26)	−0.06 (−0.16 to 0.04)
Middle SDl	32,131 (30,109–34,059)	4.20 (3.94–4.45)	29,472 (27,906–31,143)	3.93 (3.72–4.16)	−6.39 (−8.69 to −4.21)	−0.07 (−0.12 to −0.03)
Low-middle SDl	15,994 (15,170–16,792)	2.71 (2.57–2.84)	23,495 (22,277–24,800)	3.07 (2.91–3.24)	13.58 (11.87 to 15.08)	0.47 (0.43 to 0.52)
Low SDI	7339 (6979–7714)	2.62 (2.50–2.76)	17,167 (16,282–18,076)	2.94 (2.79–3.09)	11.94 (10.39 to 13.37)	0.38 (0.36 to 0.40)
Andean Latin America	557 (529–587)	2.94 (2.79–3.10)	859 (817–904)	3.63 (3.45–3.82)	23.56 (19.20 to 28.22)	0.72 (0.65 to 0.79)
Australasia	155 (144–165)	2.47 (2.30–2.64)	196 (182–209)	2.60 (2.42–2.77)	5.31 (1.22 to 9.83)	0.19 (0.14 to 0.25)
Caribbean	505 (478–532)	3.34 (3.16–3.52)	470 (448–495)	3.08 (2.94–3.25)	−7.80 (−9.89 to −5.72)	−0.26 (−0.30 to −0.23)
Central Asia	745 (710–783)	2.36 (2.25–2.48)	959 (913–1007)	2.77 (2.64–2.91)	17.34 (15.01 to 19.92)	1.10 (0.73 to 1.47)
Central Europe	975 (921–1033)	2.48 (2.34–2.63)	624 (594–657)	2.65 (2.52–2.79)	6.69 (4.46 to 9.36)	0.40 (0.31 to 0.49)
Central Latin America	4348 (4098–4581)	5.26 (4.96–5.54)	3965 (3756–4171)	4.65 (4.40–4.89)	−11.65 (−13.31 to −9.58)	−0.31 (−0.37 to −0.25)
Central Sub-Saharan Africa	768 (727–818)	2.48 (2.35–2.64)	2039 (1910–2160)	2.77 (2.60–2.94)	11.82 (8.14 to 16.07)	0.36 (0.28 to 0.43)
East Asia	25,844 (23,823–27,824)	5.62 (5.18–6.05)	15,230 (14,355–16,244)	4.42 (4.16–4.71)	−21.39 (−24.76 to −17.77)	−0.52 (−0.66 to −0.39)
Eastern Europe	2318 (2173–2466)	3.45 (3.23–3.67)	1585 (1491–1680)	3.43 (3.23–3.64)	−0.34 (−2.71 to 2.27)	0.53 (0.34 to 0.73)
Eastern Sub-Saharan Africa	3316 (3136–3493)	2.99 (2.83–3.15)	7138 (6772–7526)	3.14 (2.98–3.31)	4.91 (3.02 to 6.74)	0.12 (0.09 to 0.16)
High-income Asia Pacific	2319 (2146–2499)	4.61 (4.26–4.97)	1569 (1468–1675)	5.10 (4.77–5.44)	10.60 (7.97 to 13.24)	0.38 (0.34 to 0.41)
High-income North America	3076 (2870–3269)	3.76 (3.51–4.00)	3536 (3350–3735)	3.95 (3.74–4.17)	4.93 (2.59 to 7.83)	0.46 (0.05 to 0.87)
North Africa and Middle East	3640 (3440–3860)	2.06 (1.95–2.18)	5638 (5301–5976)	2.38 (2.24–2.53)	15.78 (13.27 to 18.24)	0.72 (0.61 to 0.82)
Oceania	80 (75–85)	2.38 (2.24–2.53)	145 (136–154)	2.27 (2.13–2.41)	−4.39 (−7.56 to −1.07)	−0.23 (−0.26 to −0.19)
South Asia	13,457 (12,745–14,182)	2.48 (2.35–2.61)	21,631 (20,417–22,940)	3.16 (2.99–3.36)	27.56 (25.49 to 29.75)	0.88 (0.81 to 0.94)
Southeast Asia	7580 (7156–7983)	3.45 (3.25–3.63)	9187 (8728–9693)	4.01 (3.81–4.23)	16.26 (14.68 to 18.14)	0.56 (0.52 to 0.61)
Southern Latin America	239 (224–256)	1.23 (1.16–1.32)	320 (300–343)	1.64 (1.54–1.76)	32.86 (27.70 to 38.14)	0.94 (0.85 to1.03)
Southern Sub-Saharan Africa	1031 (965–1095)	3.89 (3.65–4.14)	1166 (1093–1240)	3.73 (3.50–3.97)	−4.23 (−6.32 to −2.14)	−0.11 (−0.14 to −0.09)
Tropical Latin America	3202 (3031–3375)	4.62 (4.38–4.87)	1984 (1873–2107)	2.98 (2.81–3.16)	−35.55 (−37.40 to −33.62)	−1.69 (−1.88 to −1.50)
Western Europe	2022 (1892–2160)	2.06 (1.92–2.20)	2179 (2050–2322)	2.38 (2.24–2.53)	15.57 (13.17 to 18.21)	0.52 (0.48 to 0.56)
Western Sub-Saharan Africa	3883 (3678–4095)	3.61 (3.42–3.81)	9925 (9430–10,433)	3.70 (3.51–3.88)	2.31 (0.67 to 3.75)	0.10 (0.05 to 0.15)

ASR = age-standardized rate, EAPC = estimated annual percentage change, UI = uncertainty interval.

**Table 4:** The DALYs cases and age-standardized DALYs rate of paralytic ileus and intestinal obstruction in 1990 and 2021, and its temporal trends from 1990 to 2021.

Characteristics	1990		2021		1990–2021	1990–2021
	DALYs cases no. ×10^5^ (95% UI)	ASR per 100,000 no. (95% UI)	DALYs cases no. ×10^5^ (95% UI)	ASR per 100,000 no. (95% UI)	Percentage change in age-standardized rates	EAPCs of ASR
Global	3,161,663 (2,663,862–3,713,775)	139.98 (117.94–164.43)	1,613,343 (1,251,845–2,006,266)	61.21 (47.49–76.11)	–56.28 (–67.03 to –39.03)	–2.45 (–2.54 to –2.36)
High SDl	64,626 (61,037–69,222)	25.71 (24.29–27.54)	26,411 (24,157–28,650)	11.35 (10.38–12.31)	–55.87 (–59.45 to –51.85)	–2.27 (–2.40 to –2.14)
High-middle SDl	301,281 (262,420–348,900)	81.39 (70.89–94.26)	61,180 (51,405–71,755)	20.17 (16.95–23.65)	–75.22 (–80.15 to –69.61)	–4.16 (–4.29 to –4.03)
Middle SDl	1,313,623 (1,118,202–1,516,830)	171.82 (146.26–198.39)	387,956 (300,981–487,330)	51.78 (40.17–65.05)	–69.86 (–76.74 to –58.44)	–3.57 (–3.74 to –3.39)
Low-middle SDl	955,586 (7619,031–182,462)	161.68 (128.91–200.07)	531,444 (413,741–664,867)	69.53 (54.13–86.98)	–57.00 (–68.88 to –34.26)	–2.62 (–2.79 to –2.46)
Low SDI	523,996 (397,861–667,710)	187.42 (142.31–238.83)	604,811 (446,220–798,394)	103.53 (76.38–136.66)	–44.76 (–60.77 to –18.21)	–1.84 (–1.94 to –1.73)
Andean Latin America	161,583 (119,197–205,226)	852.41 (628.81–1082.64)	25,943 (19,758–33,952)	109.59 (83.46–143.42)	–87.14 (–91.10 to –80.70)	–6.81 (–7.32 to –6.30)
Australasia	713 (666–765)	11.36 (10.62–12.19)	407 (357–460)	5.40 (4.73–6.10)	–52.45 (–58.92 to –45.19)	–1.48 (–1.75 to –1.21)
Caribbean	26,628 (18,234–39,642)	176.36 (120.77–262.55)	19,784 (12,998–28,786)	129.63 (85.16–188.60)	–26.50 (–50.08 to 11.73)	–0.91 (–1.16 to –0.65)
Central Asia	72,339 (63,075–81,523)	229.07 (199.73–258.15)	33,789 (26,399–44,070)	97.59 (76.24–127.28)	–57.40 (–67.64 to –45.29)	–1.73 (–2.16 to –1.29)
Central Europe	19,758 (18,357–21,243)	50.32 (46.75–54.10)	2831 (2427–3337)	12.02 (10.30–14.17)	–76.12 (–79.86 to –70.83)	–4.03 (–4.16 to –3.90)
Central Latin America	141,229 (127,927–154,652)	170.91 (154.82–187.16)	59,471 (45,438–78,919)	69.73 (53.28–92.54)	–59.20 (–68.66 to −46.13)	–2.52 (–2.69 to –2.35)
Central Sub-Saharan Africa	53,200 (36,606–76,746)	171.68 (118.13–247.66)	62,536 (35,652–118,468)	85.01 (48.47–161.05)	−50.48 (−72.13 to −12.66)	−1.89 (−2.08 to −1.70)
East Asia	773,945 (641,046–936,230)	168.20 (139.32–203.47)	78,926 (62,607–99,211)	22.88 (18.15–28.76)	−86.40 (−89.91 to −81.95)	−6.24 (−6.46 to −6.02)
Eastern Europe	22,108 (20,583–23,695)	32.86 (30.60–35.22)	6015 (5574–6473)	13.03 (12.08–14.02)	−60.35 (−63.97 to −56.45)	−2.01 (−2.50 to −1.52)
Eastern Sub-Saharan Africa	194,614 (142,755–257,525)	175.49 (128.73–232.22)	247,893 (180,009–328,385)	108.92 (79.10–144.29)	−37.93 (−60.61 to 6.36)	−1.37 (−1.45 to −1.29)
High-income Asia Pacifc	10,307 (9277–11,651)	20.48 (18.43–23.15)	2749 (2495–3021)	8.93 (8.10–9.81)	−56.41 (−62.33 to −50.29)	−2.43 (−2.55 to −2.31)
High-income North America	20,738 (20,124–21,488)	25.37 (24.62–26.29)	13,707 (12,629–14,954)	15.31 (14.10–16.70)	−39.68 (−44.73 to −33.87)	−1.34 (−1.55 to −1.13)
North Africa and Middle East	180,327 (136,632–225,423)	102.01 (77.29–127.52)	92,513 (72,863–113,401)	39.12 (30.81–47.95)	−61.65 (−72.34 to −45.12)	−2.43 (−2.73 to −2.12)
Oceania	3159 (1955–4514)	93.85 (58.08–134.10)	1802 (1213–2520)	28.21 (18.99–39.46)	−69.94 (−83.54 to −44.73)	−4.45 (−5.10 to −3.81)
South Asia	585,325 (466,450–714,277)	107.91 (85.99–131.68)	299,299 (217,201–396,973)	43.79 (31.78–58.08)	−59.42 (−71.97 to −37.31)	−2.87 (−3.21 to −2.53)
Southeast Asia	604,584 (398,796–832,135)	274.93 (181.35–378.41)	313,573 (225,683–396,202)	136.77 (98.43–172.81)	−50.25 (−68.45 to −12.10)	−2.26 (−2.32 to −2.19)
Southern Latin America	6617 (6041–7247)	34.14 (31.17–37.40)	3652 (3158–4168)	18.72 (16.18–21.36)	−45.17 (−53.00 to −35.62)	−1.20 (−1.54 to −0.85)
Southern Sub-Saharan Africa	16,383 (13,857–19,040)	61.92 (52.37–71.95)	20,630 (16,568–24,960)	65.98 (52.99–79.83)	6.57 (−16.61 to 34.14)	0.59 (0.39 to 0.79)
Tropical Latin America	57,601 (50,145–64,526)	83.16 (72.40–93.16)	27,488 (22,335–32,562)	41.28 (33.54–48.90)	−50.36 (−61.37 to −38.07)	−1.69 (−2.04 to −1.34)
Western Europe	14,704 (14,220–15,206)	14.95 (14.46–15.46)	6477 (5919–7078)	7.06 (6.45–7.72)	−52.77 (−56.91 to −48.73)	−2.05 (−2.24 to −1.86)
Western Sub-Saharan Africa	195,800 (139,848–281,549)	182.15 (130.10–261.92)	293,858 (205,222–441,607)	109.41 (76.41–164.43)	−39.93 (−56.50 to −16.44)	−1.42 (−1.52 to −1.33)

ASR = age-standardized rate, DALYs = disability-adjusted life years, EAPC = estimated annual percentage change, UI = uncertainty interval.

### 3.2. Geographic and national variation in PI&IO burden

Marked geographic heterogeneity was observed across regions. At the regional level, the largest increases in incident cases occurred in South Asia, where cases rose from 342,875 to 551,065, and in Central Sub-Saharan Africa, where cases increased from 18,927 to 49,921. By contrast, substantial declines were observed in East Asia, where incident cases fell from 620,399 to 375,007, and in Tropical Latin America, where cases declined from 80,772 to 49,078. South Asia showed the largest increase in age-standardized incidence (PC: 27.55%; EAPC: 0.87), whereas Tropical Latin America had the greatest decline (PC: −36.80%; EAPC: −1.78). Mortality and DALY burden declined in most regions, with the most pronounced improvements in Andean Latin America and East Asia. However, some regions in Sub-Saharan Africa continued to experience increasing deaths and DALYs, and Southern Sub-Saharan Africa was the only region with rising age-standardized mortality and DALY rates (EAPC: 0.62 and 0.59, respectively). These results indicate that while major progress has been achieved in parts of East Asia and Latin America, parts of South Asia and Sub-Saharan Africa still face a persistent or worsening burden (Table 1–4, Fig. 1).

At the national level, populous countries contributed substantially to the global burden. Incident cases increased markedly in Nigeria, from 49,773 to 123,792, and in India, from 253,384 to 408,615, whereas China showed a substantial decline from 601,118 to 363,649. China also experienced a sharp reduction in severe outcomes, with deaths decreasing from 8551 to 811 and DALYs from 760,050 to 75,277. In contrast, deaths increased in Nigeria, from 903 to 1614, indicating persistent inequality in outcomes. These findings highlight pronounced national disparities in both disease occurrence and health-system performance (Fig. 1, Figure S2, Supplemental Digital Content 2).

### 3.3. Variation in burden by socio-demographic development

The burden of PI&IO varied substantially across SDI levels. In high, high-middle, and middle SDI regions, incident cases generally declined over time, whereas in low-middle SDI regions, incident cases increased from 400,376 to 579,877, and in low-SDI regions, from 180,079 to 417,812. The ASIR also increased in these lower-SDI settings, with PCs of 11.98% in low-middle SDI regions and 11.03% in low-SDI regions. Mortality patterns showed a clearer gradient across development levels. Deaths declined in all SDI groups except the low-SDI group, where deaths rose from 6094 to 7101. Although the ASMR decreased in all SDI strata, the reduction was smallest in low-SDI regions (−44.24%; EAPC: −1.82) and largest in high-middle SDI regions (−75.83%; EAPC: −4.24). A similar pattern was observed for DALYs, which increased in low-SDI regions from 523,996 to 604,811 but declined in most other SDI groups. These findings suggest that lower-resource settings continue to shoulder a disproportionate share of the burden, particularly for severe outcomes (Tables 1–4, Fig. 1).

**Figure 2.**
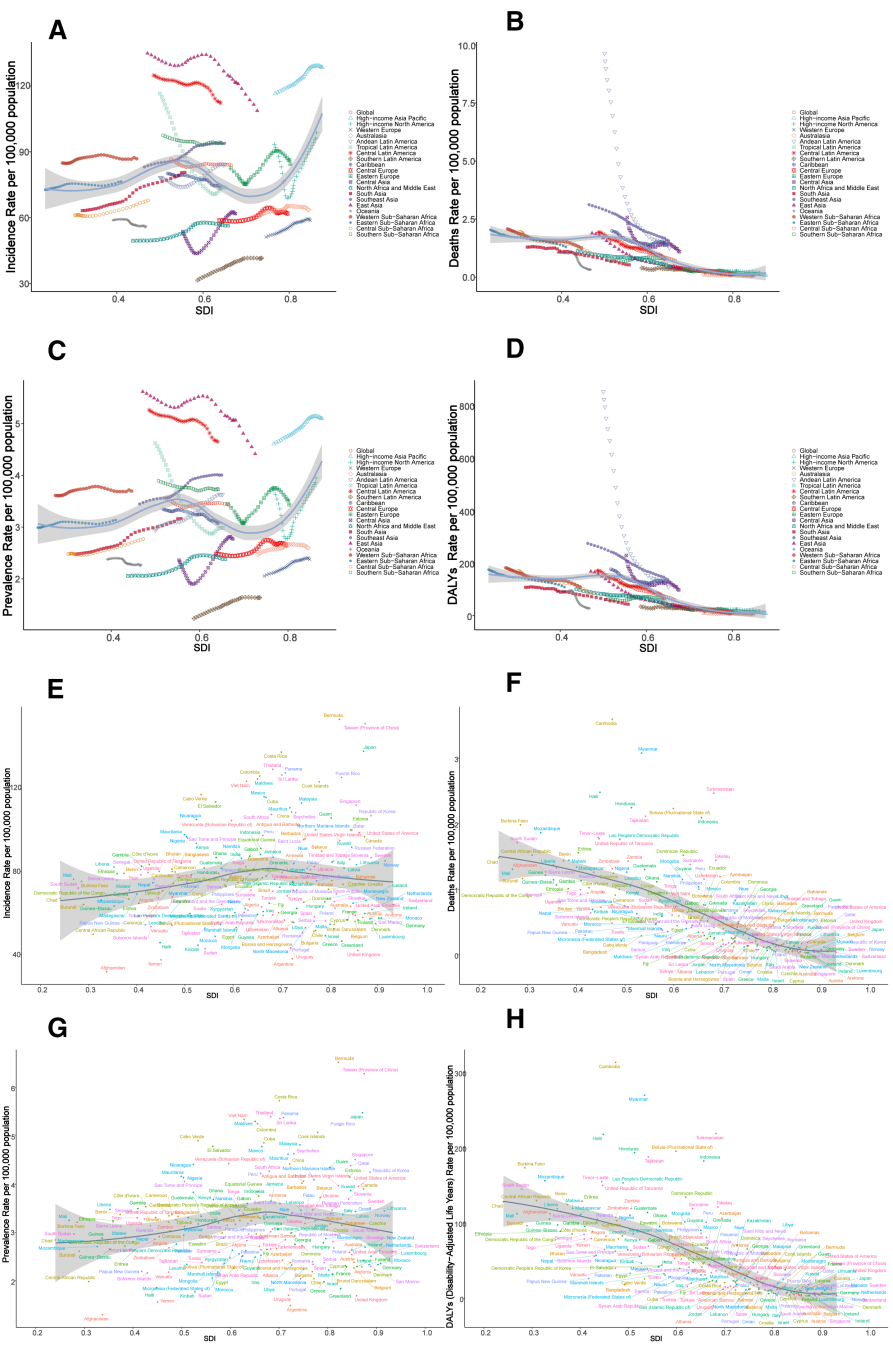
Global burden of PI&IO in different SDI regions. (A–D) Correlation between SDI and rates of incidence (A) deaths (B), prevalence (C) and DALYs (D) among 21 regions defined by GBD 2021. (E–H) Correlation between SDI and rates of incidence (E) deaths (F), prevalence (G), and DALYs (H) among 204 countries. Spearman correlation analysis was applied to measure the *r* indices and *P*-values for the association of ASR with SDI. DALYs = disability-adjusted life years, GBD 2021 = Global Burden of Disease 2021, PI&IO = paralytic ileus and intestinal obstruction, SDI = socio-demographic index.

Correlation analyses supported this pattern. SDI was not significantly associated with the age-standardized incidence rate (*R* = 0.0194, *P* = .608) or prevalence rate (*R* = 0.0208, *P* = .581), but showed strong negative correlations with both the mortality rate (*r* = −0.7844, *P* < .001) and DALY rate (*r* = −0.7783, *P* < .001). Thus, socioeconomic development appeared to be more strongly related to the risk of death and disability than to disease occurrence itself (Fig. 2).

### 3.4. Age-specific patterns of PI&IO burden

**Figure 3.**
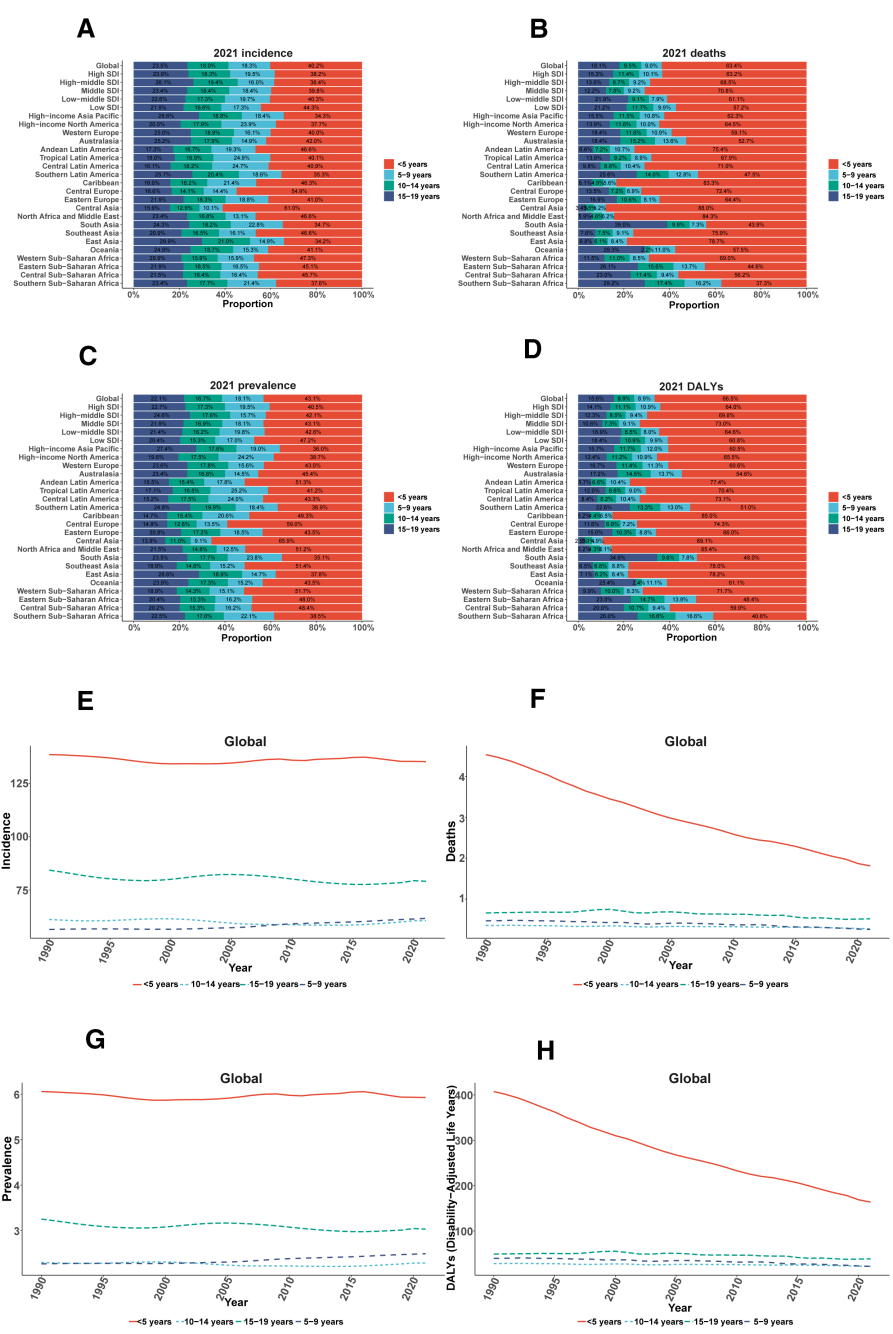
Temporal and spatial distribution of global burden of PI&IO among different age groups. (A–D) Composition ratio of incidence (A) deaths (B), prevalence (C) and DALYs (D) among different age groups globally and regionally in 2021. (E–H) Trends of incidence (E) deaths (F), prevalence (G), and DALYs (H) of PI&IO by age groups from 1990 to 2021. DALYs = disability-adjusted life years, PI&IO = paralytic ileus and intestinal obstruction.

Children younger than 5 years consistently bore the highest burden of PI&IO across all major indicators in 2021. Globally, this age group had the highest incidence rate (135.26 per 100,000), mortality rate (1.81 per 100,000), prevalence rate (5.93 per 100,000), and DALY rate (163.58 per 100,000). Within this youngest age group, regional differences were pronounced. Mortality and DALY rates were highest in the Caribbean (4.85 and 435.65 per 100,000, respectively) and Southeast Asia (4.81 and 432.18 per 100,000, respectively). Prevalence showed a U-shaped age pattern, with a secondary peak among adolescents aged 15 to 19 years (3.03 per 100,000 globally), particularly in East Asia and High-income Asia Pacific. These findings indicate that both early childhood and late adolescence are important periods for disease burden, although the most severe burden remains concentrated in the youngest children (Fig. 3A–D).

Temporal analysis further showed that mortality and DALYs declined across all age groups, with the greatest improvement among children younger than 5 years. In this age group, mortality decreased by 60.1%, from 4.54 to 1.81 per 100,000, and DALYs declined by 59.9%, from 407.47 to 163.58 per 100,000. Socioeconomic disparities were also striking: mortality in low-SDI regions was 8.3 times that in high-SDI regions, and the DALY burden among children under 5 years was nearly 8-fold higher in low-SDI settings. These results underscore that early childhood remains the most vulnerable period for PI&IO burden worldwide (Fig. 3E–H).

### 3.5. Temporal trends and age–period–cohort effects

**Figure 4.**
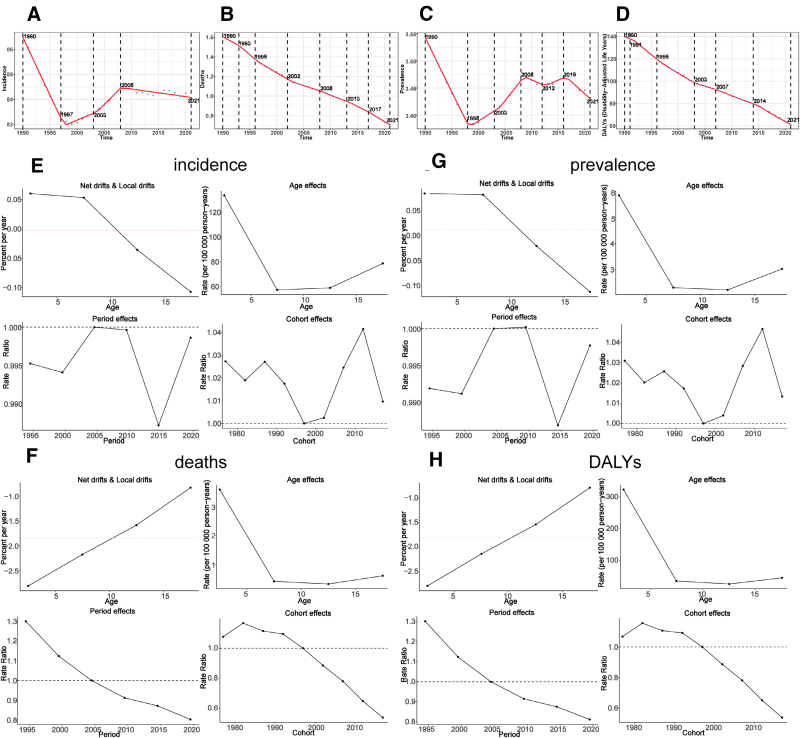
Join point regression model analysis and age–period–cohort analysis to estimate the temporal trend of global burdens of PI&IO. (A–D) Line plot of AAPC for incidence (A) deaths (B), prevalence (C) and DALYs (D) of PI&IO from 1990 to 2021. (E–H) Age–period–cohort analysis of PI&IO in incidence (E) deaths (F), prevalence (G), and DALYs (H). Net and local drifts >0 indicate that the burden of PI&IO is rising (top left); the impact of age effects on the burden of PI&IO (top right); a rate ratio of period and cohort effects >1 indicates an increased burden of PI&IO (bottom panel). AAPC = average annual percentage change, DALYs = disability-adjusted life years, PI&IO = paralytic ileus and intestinal obstruction.

Joinpoint regression showed modest long-term changes in incidence and prevalence, but substantial declines in mortality and DALYs. Globally, incidence decreased slightly overall, with an AAPC of −0.08% (95% CI: −0.10% to −0.05%), whereas mortality declined more markedly, with an AAPC of −2.61% (95% CI: −2.74% to −2.48%). Prevalence also decreased slightly (AAPC: −0.10%, 95% CI: −0.12% to −0.08%), while DALYs showed a pronounced decline (AAPC: −2.70%, 95% CI: −2.81% to −2.58%). The steepest recent reductions were observed for mortality and DALYs after 2015. These results suggest that the occurrence of PI&IO has remained relatively stable, whereas severe outcomes have improved substantially over time (Fig. 4A–D).

The age–period–cohort analysis demonstrated distinct age effects across indicators. Incidence showed a J-shaped age pattern, with the highest burden in early childhood, a decline through mid-childhood, and a rise again in late adolescence. Prevalence and DALYs showed U-shaped patterns. For mortality and DALYs, both period and cohort effects declined significantly, and later birth cohorts had markedly lower risks than earlier ones. By contrast, period and cohort effects were not significant for incidence and prevalence. Together, these findings indicate that age remains the dominant determinant of disease occurrence, whereas improvements in healthcare and broader social conditions have mainly reduced fatal and disabling outcomes (Fig. 4E–H).

### 3.6. Contributions of demographic and epidemiological factors

**Figure 5.**
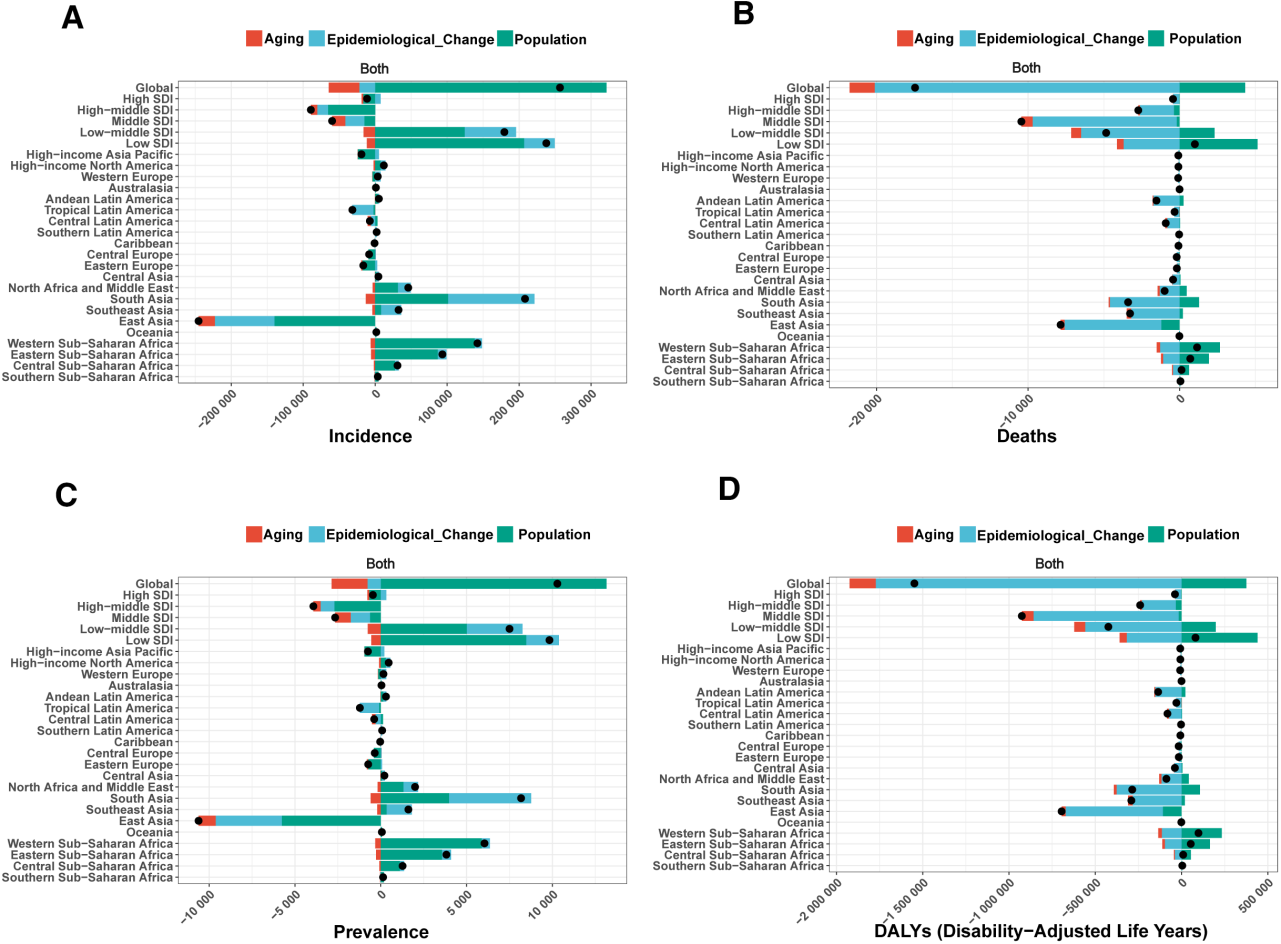
Decomposition analysis for PI&IO. The figure presents the analysis for incidence (A) deaths (B), prevalence (C) and DALYs (D). DALYs = disability-adjusted life years, PI&IO = paralytic ileus and intestinal obstruction.

Decomposition analysis revealed that different mechanisms underlay changes in different burden indicators. The increase in incident cases was primarily driven by population growth. Globally, the net increase of 256,738 incident cases was largely attributable to population growth (+125.17%), partly offset by epidemiological improvement (−8.51%) and population aging (−16.66%). Similarly, the increase in prevalent cases (+10,288) was mainly driven by population growth (+127.82%). In contrast, epidemiological improvement accounted for most of the decline in severe outcomes: it explained +115.20% of the net reduction in deaths and +114.41% of the reduction in DALYs, despite being partly offset by population growth. This pattern was especially evident in most higher-performing regions, whereas in parts of Sub-Saharan Africa, rapid population growth offset much of the epidemiological gain. These findings suggest that population growth is the main driver of increasing case numbers, while improved disease management and healthcare likely underlie the decline in mortality and DALYs (Fig. 5A–D).

### 3.7. Inequality in PI&IO burden

**Figure 6.**
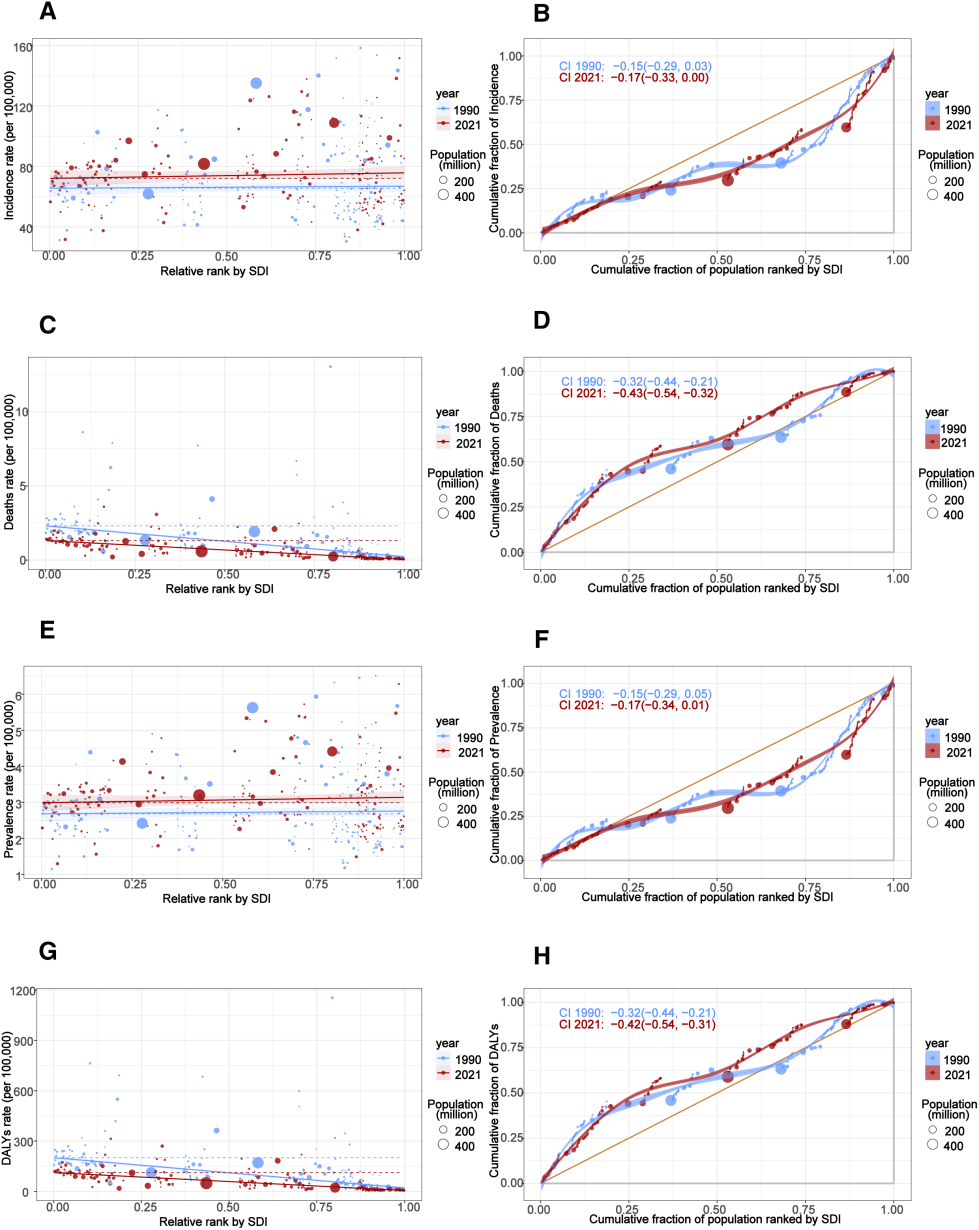
Regional disparities for the disease burden of PI&IO. (A–H) Worldwide health inequality regression curves and concentration curves for the DALYs of PI&IO in (1990, 2021) respectively. The relationship between SDI and ASIR (A) ASMR (C) ASPR (E) and ASDR (G) depicted by the slope index of inequality. The relative inequalities depicted by the concentration index of ASIR (B), ASMR (D), ASPR (F), and ASDR (H). Blue represents data in 1990, and red represents data in 2021. ASDR = age-standardized DALY rate, ASIR = age-standardized incidence rates, ASMR = age-standardized deaths rate, ASPR = age-standardized prevalence rate, ASR = age-standardized rate, DALYs = disability-adjusted life years, PI&IO = paralytic ileus and intestinal obstruction, SDI = socio-demographic index.

Inequality analyses showed distinct patterns across burden measures. Incidence and prevalence were distributed relatively evenly across development levels, and the corresponding inequality estimates were not statistically robust. In contrast, mortality and DALYs exhibited persistent and significant pro-poor inequality. For mortality, the SII remained significantly negative in both 1990 (−2.08, 95% CI: −2.29 to −1.87) and 2021 (−1.26, 95% CI: −1.37 to −1.16), suggesting that the absolute gap narrowed over time. However, the CII became more negative, from −0.324 to −0.428, indicating worsening relative inequality. A similar pattern was observed for DALYs, with the SII improving from −179.98 to −106.72 but the CII remaining strongly negative and worsening over time (from −0.32 to −0.42). These findings indicate that although absolute inequalities in severe outcomes have narrowed, the relative burden remains concentrated in poorer populations (Fig. 6A–H).

### 3.8. Frontier analysis

**Figure 7.**
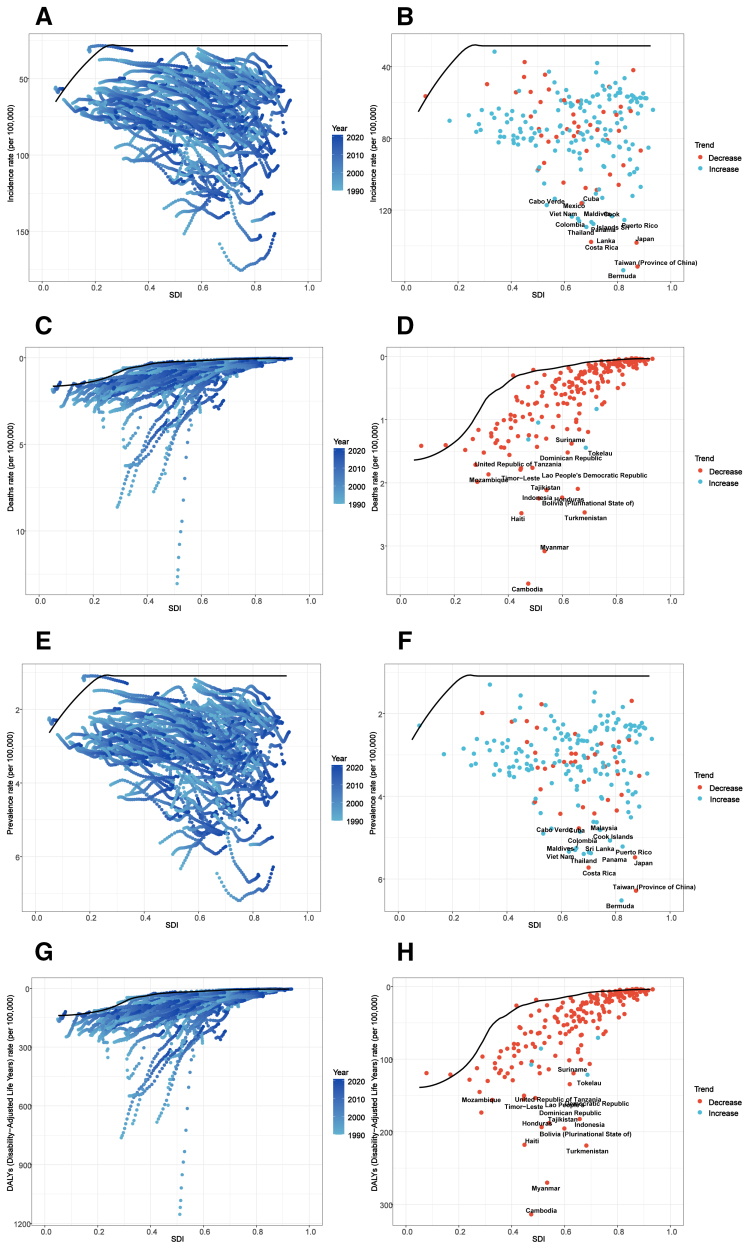
Frontier analysis of the SDI and the global burden of PI&IO. Based on data from 204 countries and territories, the figure visualizes the association between SDI and 4 age-standardized burden metrics: ASIR (A, B), ASMR (C, D), ASPR (E, F), and ASDR (G, H). ASDR = age-standardized DALY rate, ASIR = age-standardized incidence rates, ASMR = age-standardized deaths rate, ASR = age-standardized rate, DALYs = disability-adjusted life years, PI&IO = paralytic ileus and intestinal obstruction, SDI = socio-demographic index.

Frontier analysis showed that development status alone did not fully explain performance. For incidence and prevalence, the largest efficiency gaps were mainly found in high- and high-middle SDI settings, including Bermuda, Taiwan (Province of China), Japan, and Costa Rica, indicating considerable avoidable burden despite relatively favorable socioeconomic conditions. By contrast, the smallest gaps were observed in several low-SDI countries such as Somalia and Afghanistan. For mortality and DALYs, however, the largest gaps were concentrated in low- and middle SDI countries, including Cambodia, Myanmar, Turkmenistan, and Haiti, suggesting substantial room for improvement in severe outcomes. Notably, several low-SDI countries, including Bangladesh, Somalia, Niger, and Papua New Guinea, performed at or near their theoretical frontier for mortality and DALYs. These findings suggest that high SDI does not necessarily guarantee optimal performance, whereas some resource-limited settings have achieved relatively efficient outcomes under constrained conditions (Fig. 7A–H).

### 3.9. Forecasted burden through 2050

**Figure 8.**
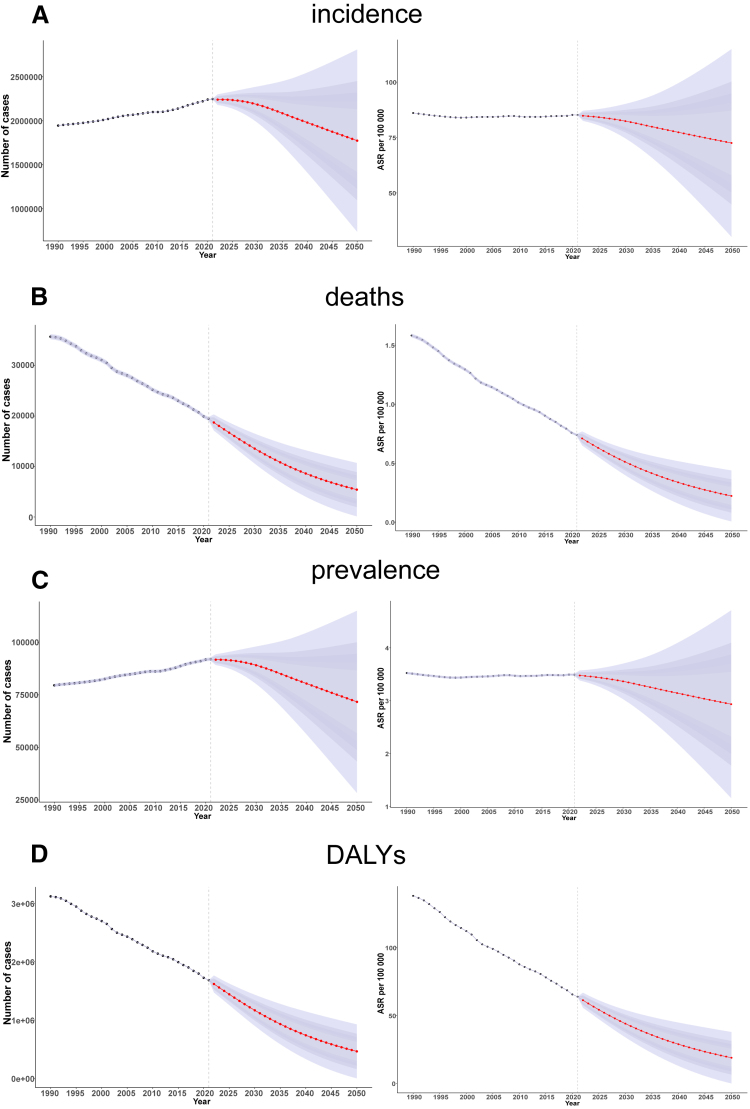
Prediction of the disease burden trend of PI&IO from 1990 to 2050. (A) Prediction of PI&IO in all age cases (left) and ASR (right) of incidence (A) deaths (B), prevalence (C) and DALYs (D) till 2050 by using the BAPC model. ASR = age-standardized rate, BAPC = Bayesian age–period–cohort, DALYs = disability-adjusted life years, PI&IO = paralytic ileus and intestinal obstruction.

The Bayesian age–period–cohort projections indicate that the absolute number of incident cases will continue to increase through 2050, rising from approximately 2.21 million in 2021 to about 2.41 million in 2050. However, the age-standardized incidence rate is expected to remain broadly stable, peaking at approximately 84.85 per 100,000 around 2038 and then stabilizing near 83.93 per 100,000 by 2050. In contrast, severe outcomes are projected to continue improving. Deaths are expected to decrease from 18,728 in 2021 to approximately 9961 in 2050, with the ASMR declining from 0.71 to 0.33 per 100,000. DALYs are also projected to fall substantially, from 1.61 million in 2021 to approximately 0.84 million in 2050, and the age-standardized DALY rate is expected to decline from 61.2 to 27.7 per 100,000. Prevalence is projected to remain relatively stable, with only a slight decline in case numbers by 2050. Overall, these forecasts suggest continued improvement in mortality and disability burden, but persistent healthcare demand because of the growing number of incident cases (Fig. 8A–D).

## 4. Discussion

This study provides a comprehensive assessment of the global burden of PI&IO among individuals younger than 20 years from 1990 to 2021 using data from the GBD Study 2021. Several key findings emerged. First, although age-standardized rates of incidence, mortality, prevalence, and DALYs generally declined over time, the absolute number of incident and prevalent cases increased globally. Second, the burden of PI&IO was concentrated disproportionately in children under 5 years of age and in lower-SDI settings. Third, marked inequalities persisted, especially for mortality and DALYs, indicating that severe outcomes remain heavily concentrated in disadvantaged regions. Finally, decomposition and age–period–cohort analyses suggested that reductions in mortality and DALYs were mainly driven by epidemiological improvement, whereas increases in case numbers were largely attributable to population growth.^[3,29]^

One important finding is the divergence between declining age-standardized rates and increasing absolute case numbers. This pattern is consistent with previous GBD-based studies showing that demographic expansion may offset epidemiological improvement and sustain or even increase healthcare demand despite declining rates. From a policy perspective, this distinction is important. Declining age-standardized mortality or DALY rates may indicate progress in prevention and care, but increasing absolute case numbers still imply growing demand for pediatric emergency evaluation, hospital care, surgery, and perioperative support. Therefore, both standardized rates and absolute counts should be considered when assessing the burden of PI&IO and planning health services.^[4,6,30]^

Our findings also highlight the particular vulnerability of children under 5 years of age, who consistently showed the highest rates of incidence, mortality, prevalence, and DALYs. This age-related pattern is clinically plausible and agrees with previous pediatric surgical literature, which shows that younger children are more susceptible to severe progression because symptoms may be nonspecific, physiological reserve is limited, and delays in caregiver recognition or referral can rapidly worsen outcomes. In lower-resource settings, these challenges may be compounded by barriers to timely diagnosis, pediatric anesthesia, specialist consultation, and emergency surgical care. These observations suggest that efforts to improve early recognition, rapid referral, and age-appropriate emergency management should prioritize younger children.^[31–34]^

Another notable finding is the persistence of inequality across socio-demographic settings. Although incidence and prevalence showed relatively modest inequality, mortality and DALY burdens remained disproportionately concentrated in lower-SDI regions. This suggests that socioeconomic disadvantage may affect outcomes more strongly than disease occurrence itself. Children in low-resource settings may not only experience delayed diagnosis and treatment, but may also have reduced access to safe surgery, perioperative care, and postoperative support, resulting in a much greater risk of severe complications, disability, and death. This interpretation is consistent with previous research in global surgery and health inequality, which emphasizes the critical role of health-system capacity, workforce availability, referral efficiency, and equitable access to emergency care in determining surgical outcomes.^[27,35,36]^

The frontier analysis adds a further dimension to these findings. Some lower-SDI countries performed close to their expected frontier for mortality and DALYs, whereas several higher-SDI settings still showed avoidable gaps in burden relative to their development level. This suggests that socioeconomic development alone does not guarantee optimal outcomes and that health-system organization and efficiency remain important. Such findings are valuable because they highlight not only where the burden is high, but also where outcomes may be improved under existing resource constraints.^[37–39]^

Our decomposition results further clarify the mechanisms underlying changing burden patterns. Population growth was the major contributor to increasing incident and prevalent cases, whereas epidemiological improvement explained most of the reductions in deaths and DALYs. This indicates that gains in clinical management and healthcare quality may already be reducing severe outcomes, but that these benefits are partly offset by growing pediatric populations in many regions. Similar patterns have been reported in other disease burden analyses, in which demographic growth continues to drive rising absolute numbers even when rates improve.^[40–43]^

Our findings should be interpreted in the context of the recent study by Zhao et al,^[8]^ which described the global burden of PI&IO in the overall population using GBD 2021 data. While their study provided valuable evidence on incidence and YLLs across all ages, the present study differs in several important aspects. First, we focused specifically on individuals younger than 20 years, thereby addressing the pediatric and adolescent burden of PI&IO. Second, we assessed a broader range of indicators, including incidence, mortality, prevalence, and DALYs. Third, we further applied Joinpoint regression, age–period–cohort analysis, decomposition analysis, inequality analysis, frontier analysis, and BAPC forecasting. These methodological extensions enabled us to identify the drivers of changing burden, persistent socioeconomic inequalities, performance gaps across SDI levels, and projected future patterns among children and adolescents. Thus, compared with previous studies, the present analysis provides a broader and more population-specific perspective, offering complementary evidence for targeted pediatric surgical care and equity-oriented public health strategies.^[44–46]^

These findings have several practical implications. In higher-SDI settings, further efforts may focus on prevention, early recognition, and reduction of residual avoidable morbidity. In lower-SDI settings, priority should be given to strengthening emergency surgical systems, including referral pathways, workforce training, transport, pediatric anesthesia, and perioperative care. Because children under 5 years bear the heaviest burden, interventions should be specifically designed for this age group.^[47]^

This study has several strengths, including the use of standardized GBD 2021 estimates across 204 countries and territories over more than 3 decades and the application of multiple complementary analytical approaches. However, limitations should also be acknowledged. The study is based on modeled GBD estimates rather than individual-level clinical data, and PI&IO was analyzed as a broad category without detailed etiological stratification. In addition, patient-level factors such as referral delay, disease severity, operative timing, and perioperative management could not be directly assessed.^[48]^

Future research should include multicenter prospective studies to validate these population-level findings, incorporate individual-level clinical data, and better define how etiology, referral delay, diagnostic capacity, operative timing, and perioperative care influence outcomes. Such studies would help translate burden estimates into more targeted and clinically actionable strategies.

In conclusion, PI&IO remains an important cause of pediatric disease burden worldwide. Although age-standardized rates have declined, persistent inequality and increasing absolute case numbers continue to challenge health systems, particularly in lower-SDI settings and among children under 5 years of age. Addressing these challenges will require not only continued improvements in clinical care, but also more equitable strengthening of pediatric emergency surgical systems worldwide.^[49]^

## 5. Conclusions

This study delineates a global burden of pediatric PI&IO characterized by declining age-standardized rates yet rising absolute case numbers. While mortality and DALYs have decreased substantially due to care improvements, significant and persistent socioeconomic inequalities remain, particularly for severe outcomes. Population growth is the main driver of increasing incident cases. Public health strategies must be tailored: high-SDI regions should optimize prevention to reduce incidence, while low-SDI regions must strengthen acute care systems to sustain mortality reductions, with all efforts prioritizing children under 5.

## Acknowledgments

This study was generously supported by Jingding Medical Tech, to whom we extend our sincere gratitude. We especially thank them for providing authorization and technical support for the JD_GBDR software. The team at Jingding Medical Tech offered invaluable assistance in data processing.

## Author contributions

**Conceptualization:** Meilin Zhang.

**Data curation:** Meilin Zhang.

**Formal analysis:** Fangbing Li.

**Investigation:** Yaohui Dong.

**Methodology:** He Yang.

**Project administration:** Yaohui Dong.

**Resources:** He Yang.

**Software:** Huina Yu.

**Supervision:** Fangbing Li.

**Visualization:** Xia Yan.

**Writing – original draft:** Huina Yu.

**Writing – review & editing:** Xia Yan.





## References

[1] Peyvasteh M Askarpour S Javaherizadeh H Taghizadeh S. Ileus and intestinal obstruction – comparison between children and adults. Pol Przegl Chir. 2011;83:367–71. 22166664 10.2478/v10035-011-0058-9

[2] Kliegman RM Walker WA Yolken RH. Necrotizing enterocolitis: research agenda for a disease of unknown etiology and pathogenesis. Pediatr Res. 1993;34:701–8. 8108179 10.1203/00006450-199312000-00001

[3] Ferrari AJ Santomauro DF Aali A. Global incidence, prevalence, years lived with disability (YLDs), disability-adjusted life-years (DALYs), and healthy life expectancy (HALE) for 371 diseases and injuries in 204 countries and territories and 811 subnational locations, 1990–2021: a systematic analysis for the Global Burden of Disease Study 2021. Lancet. 2024;403:2133–61. 38642570 10.1016/S0140-6736(24)00757-8 PMC11122111

[4] Hu X Lin X Dai Z Fang K. Global burden of pediatric fracture (1992–2021) and projections of future disease burden trends. BMC Pediatr. 2025;25:416. 40413433 10.1186/s12887-025-05767-6 PMC12102943

[5] Poenaru D Pemberton J Cameron BH. The burden of waiting: DALYs accrued from delayed access to pediatric surgery in Kenya and Canada. J Pediatr Surg. 2015;50:765–70. 25783371 10.1016/j.jpedsurg.2015.02.033

[6] James SL Abate D Abate KH. Global, regional, and national incidence, prevalence, and years lived with disability for 354 diseases and injuries for 195 countries and territories, 1990–2017: a systematic analysis for the Global Burden of Disease Study 2017. Lancet. 2018;392:1789–858. 30496104 10.1016/S0140-6736(18)32279-7 PMC6227754

[7] Athukorala S Kansara N Lehman E Pantanelli SM. Global health spending set to grow, but remain unequally distributed between countries. PharmacoEcon Outcomes News. 2021;888:12. 34611392 10.1007/s40274-021-08062-y PMC8484845

[8] Zhao YC Zhang H Wu S. Global burden of paralytic ileus and intestinal obstruction, 1990–2021: a GBD 2021 analysis. J Health Popul Nutr. 2025;44:294. 40826126 10.1186/s41043-025-01045-4 PMC12362850

[9] Wang L Mi Y Zhu X. Global, regional, and National burden of falls among midlife women from 1990 to 2021 and projections to 2050: a systematic analysis for the global burden of disease study 2021. Aging Clin Exp Res. 2025;37:324. 41236661 10.1007/s40520-025-03210-5 PMC12618396

[10] Santos AC Willumsen J Meheus F Ilbawi A Bull FC. The cost of inaction on physical inactivity to public health-care systems: a population-attributable fraction analysis. Lancet Glob Health. 2023;11:e32–9. 36480931 10.1016/S2214-109X(22)00464-8 PMC9748301

[11] Ding D Lawson KD Kolbe-Alexander TL. The economic burden of physical inactivity: a global analysis of major non-communicable diseases. Lancet. 2016;388:1311–24. 27475266 10.1016/S0140-6736(16)30383-X

[12] Vos T Lim SS Abbafati C. Global burden of 369 diseases and injuries in 204 countries and territories, 1990–2019: a systematic analysis for the Global Burden of Disease Study 2019. Lancet. 2020;396:1204–22. 33069326 10.1016/S0140-6736(20)30925-9 PMC7567026

[13] Longwu X Teng X. A socioeconomic perspective on the global burden of gastroesophageal reflux disease (1990–2021): findings from the GBD 2021. BMC Gastroenterol. 2025;25:794. 41214515 10.1186/s12876-025-04389-7 PMC12604397

[14] Gu H Han K Li J. Analysis of the disease burden of vertebral fractures in China and worldwide from 1990 to 2021 and trend forecast to 2035. J Health Popul Nutr. 2025;44:378. 41152965 10.1186/s41043-025-01114-8 PMC12570628

[15] Zheng C Huang H Zhang L. The burden of low back pain and predictions in Asia-Pacific region, 1990–2021: a comparative analysis of China, Japan, Thailand, and Pakistan. Front Med (Lausanne). 2026;13:1693067. 41716806 10.3389/fmed.2026.1693067 PMC12913483

[16] Trinidad S Goldshore M Kotagal M. Addressing health equity in pediatric surgical care in the United States – progress and challenges. Semin Pediatr Surg. 2023;32:151354. 37967486 10.1016/j.sempedsurg.2023.151354

[17] von Elm E Altman DG Egger M Pocock SJ Gøtzsche PC Vandenbroucke JP. The strengthening the reporting of observational studies in epidemiology (STROBE) statement: guidelines for reporting observational studies. PLoS Med. 2007;4:e296. 17941714 10.1371/journal.pmed.0040296 PMC2020495

[18] Maphosa T Denoeud-Ndam L Chilikutali L. Impact of an optimized care model for advanced HIV disease: a non-randomized cluster study in Malawi. BMC Public Health. 2025;25:2802. 40819079 10.1186/s12889-025-24157-2 PMC12357363

[19] Zhang K Kan C Han F. Global, regional, and national epidemiology of diabetes in children from 1990 to 2019. JAMA Pediatr. 2023;177:837–46. 37399036 10.1001/jamapediatrics.2023.2029 PMC10318549

[20] Clegg LX Hankey BF Tiwari R Feuer EJ Edwards BK. Estimating average annual per cent change in trend analysis. Stat Med. 2009;28:3670–82. 19856324 10.1002/sim.3733 PMC2843083

[21] Muggeo VM. Comment on ‘Estimating average annual per cent change in trend analysis’ by Clegg LX, Hankey BF, Tiwari R, Feuer EJ, Edwards BK, Statistics in Medicine 2009; 28:3670–3682. Stat Med. 2010;29:1958–60; author reply 1961. 20680988 10.1002/sim.3850

[22] Zou Z Cini K Dong B. Time trends in cardiovascular disease mortality across the BRICS: an age–period–cohort analysis of key nations with emerging economies using the Global Burden of Disease Study 2017. Circulation. 2020;141:790–9. 31941371 10.1161/CIRCULATIONAHA.119.042864

[23] Zhang J Pan L Guo Q. The impact of global, regional, and national population ageing on disability-adjusted life years and deaths associated with diabetes during 1990–2019: a global decomposition analysis. Diabetes Metab Syndr. 2023;17:102791. 37271078 10.1016/j.dsx.2023.102791

[24] Jiang CY Han K Yang F. Global, regional, and national prevalence of hearing loss from 1990 to 2019: a trend and health inequality analyses based on the Global Burden of Disease Study 2019. Ageing Res Rev. 2023;92:102124. 37972859 10.1016/j.arr.2023.102124

[25] Pan H Zhao Z Deng Y. The global, regional, and national early-onset colorectal cancer burden and trends from 1990 to 2019: results from the Global Burden of Disease Study 2019. BMC Public Health. 2022;22:1896. 36221047 10.1186/s12889-022-14274-7 PMC9555189

[26] Han Y Hao G Chu G. Global, regional and national burdens of epilepsy in the adolescents and young adults from 1990 to 2021 and its predictions. BMC Neurol. 2025;25:402. 41029475 10.1186/s12883-025-04331-0 PMC12487120

[27] Lu H Cheng X Xiong J. The global burden of adverse effects of medical treatment: a 30-year socio-demographic and geographic analysis using GBD 2021 data. Front Big Data. 2025;8:1590551. 40821361 10.3389/fdata.2025.1590551 PMC12354518

[28] Feng L Wang Y Li L Wang X Feng J. Global burden of HIV and drug-resistant tuberculosis co-infection and its attributable risk factors, 1990 to 2021, with projections to 2031. BMC Infect Dis. 2025;25:1521. 41204126 10.1186/s12879-025-11830-5 PMC12595808

[29] Hu T Yang X Du Y. Trends in the global, regional, and national burden of cardiovascular diseases attributed to high systolic blood pressure from 1990 to 2021 and projections to 2045: a systematic analysis based on GBD 2021 data. BMC Cardiovasc Disord. 2025;25:390. 40399813 10.1186/s12872-025-04807-4 PMC12096714

[30] Zhang Y Huang S Wang Y. Temporal trends and geographical variations in pediatric urinary tract infections: a comprehensive analysis using the global burden of disease study 2021. Trop Med Health. 2025;53:170. 41287076 10.1186/s41182-025-00829-y PMC12642102

[31] Zhou J Zhu J Zhang P Tao C Hong X Zhang Z. Global, regional, and national burdens of Clostridioides difficile infection over recent decades: a trend analysis informed by the Global Burden of Disease Study. Microbiol Spectr. 2025;13:e0129024. 40272190 10.1128/spectrum.01290-24 PMC12131777

[32] Wu Y Zhang X Lin Z. Changes in the global burden of foreign body aspiration among under-5 children from 1990 to 2019. Front Pediatr. 2023;11:1235308. 37727616 10.3389/fped.2023.1235308 PMC10506258

[33] Hendel S Coonan T Thomas S McQueen K. The rate-limiting step: the provision of safe anesthesia in low-income countries. World J Surg. 2014;39:833–41. 10.1007/s00268-014-2775-9 25201470

[34] Ahmed MM Oweidat M Okesanya OJ. Barriers to pediatric emergency care in low-resource settings: a narrative review. Sage Open Pediatr. 2025;12:1–9. 10.1177/30502225251336861 PMC12220907 40612221

[35] Yu Y Li H Hu NX. Global burden and health inequality of nutritional deficiencies from 1990 to 2019. Front Nutr. 2024;11:1470713. 39385781 10.3389/fnut.2024.1470713 PMC11461340

[36] Ademuyiwa AO Hardy P Runigamugabo E. Reducing surgical site infections in low-income and middle-income countries (FALCON): a pragmatic, multicentre, stratified, randomised controlled trial. Lancet. 2021;398:1687–99. 34710362 10.1016/S0140-6736(21)01548-8 PMC8586736

[37] Chen C Wang L Du S Qiu Y Liu Y Teng Q. Global burden of chronic lymphocytic leukemia from 1990 to 2021. Cancer Control. 2025;32:10732748251397071. 41223053 10.1177/10732748251397071 PMC12612519

[38] Murray CJL Aravkin AY Zheng P. Global burden of 87 risk factors in 204 countries and territories, 1990–2019: a systematic analysis for the Global Burden of Disease Study 2019. Lancet. 2020;396:1223–49. 33069327 10.1016/S0140-6736(20)30752-2 PMC7566194

[39] He Y Zhou M Tang J. Global, regional, and national burden of lower extremity peripheral arterial disease: a systematic analysis of prevalence, incidence, deaths, and dalys from 1990 to 2021 with projections and dietary risk factors to 2036. J Health Popul Nutr. 2025;44:351. 41063331 10.1186/s41043-025-01094-9 PMC12505598

[40] Huixian L Wanhong L Ani W Hongli J Jun L. Changing epidemiology of chronic kidney disease as a result of type 2 diabetes mellitus from 1990 to 2017: estimates from Global Burden of Disease 2017. J Diabetes Investig. 2020;12:346–56. 10.1111/jdi.13355 PMC7926234 32654341

[41] Liping H Hui L Chen G Wenqiang Y Mingxing Y. Global burden, regional disparities, and decomposition analysis of metabolic risk-attributable diseases among women of reproductive age: GBD 2021. BMC Womens Health. 2025;25:474. 41053863 10.1186/s12905-025-04005-6 PMC12502237

[42] Li H Song X Liang Y. Global, regional, and national burden of disease study of atrial fibrillation/flutter, 1990–2019: results from a global burden of disease study, 2019. BMC Public Health. 2022;22:2015. 36329400 10.1186/s12889-022-14403-2 PMC9632152

[43] Xie J Li W Li X. Global, regional, and national epidemiology of type 1 diabetes in children from 1990 to 2021: trend and health inequality analyses based on the Global Burden of Disease Study 2021. Diabetol Metab Syndr. 2025;17:337. 40826118 10.1186/s13098-025-01905-3 PMC12359914

[44] Liu Y Yang X He Z. Spinal cord injury: global burden from 1990 to 2019 and projections up to 2030 using Bayesian age–period–cohort analysis. Front Neurol. 2023;14:1304153. 38116113 10.3389/fneur.2023.1304153 PMC10729761

[45] Su Z Zou Z Hay SI. Global, regional, and national time trends in mortality for congenital heart disease, 1990–2019: an age–period–cohort analysis for the Global Burden of Disease 2019 study. EClinicalMedicine. 2022;43:101249. 35059612 10.1016/j.eclinm.2021.101249 PMC8760503

[46] Ren Y Xu R Wang Y Su L Su J. Global, regional, and national burden of ovarian cancer in women aged 45 + from 1990 to 2021 and projections for 2050: a systematic analysis based on the 2021 global burden of disease study. J Cancer Res Clin Oncol. 2025;151:225. 40751826 10.1007/s00432-025-06277-9 PMC12317933

[47] Wright N Jensen G St‐Louis E. Global initiative for children’s surgery: a model of global collaboration to advance the surgical care of children. World J Surg. 2019;43:1416–25. 30623232 10.1007/s00268-018-04887-8 PMC7019676

[48] Wu Z Xia F Lin R. Global burden of cancer and associated risk factors in 204 countries and territories, 1980–2021: a systematic analysis for the GBD 2021. J Hematol Oncol. 2024;17:119. 39614359 10.1186/s13045-024-01640-8 PMC11607901

[49] Kassebaum N Kyu HH Zoeckler L. Child and adolescent health from 1990 to 2015: findings from the global burden of diseases, injuries, and risk factors 2015 study. JAMA Pediatr. 2017;171:573–92. 28384795 10.1001/jamapediatrics.2017.0250 PMC5540012

