# Supplementary figures and images for: Global, regional, and national disease burden of paralytic ileus and intestinal obstruction among individuals younger than 20 years, 1990 to 2021: Findings from the Global Burden of Disease Study 2021
Source: Medicine (Baltimore). 2026 Jul 10;105(28):e49668. doi: 10.1097/MD.0000000000049668 (PMC13363197; doi:10.1097/MD.0000000000049668)

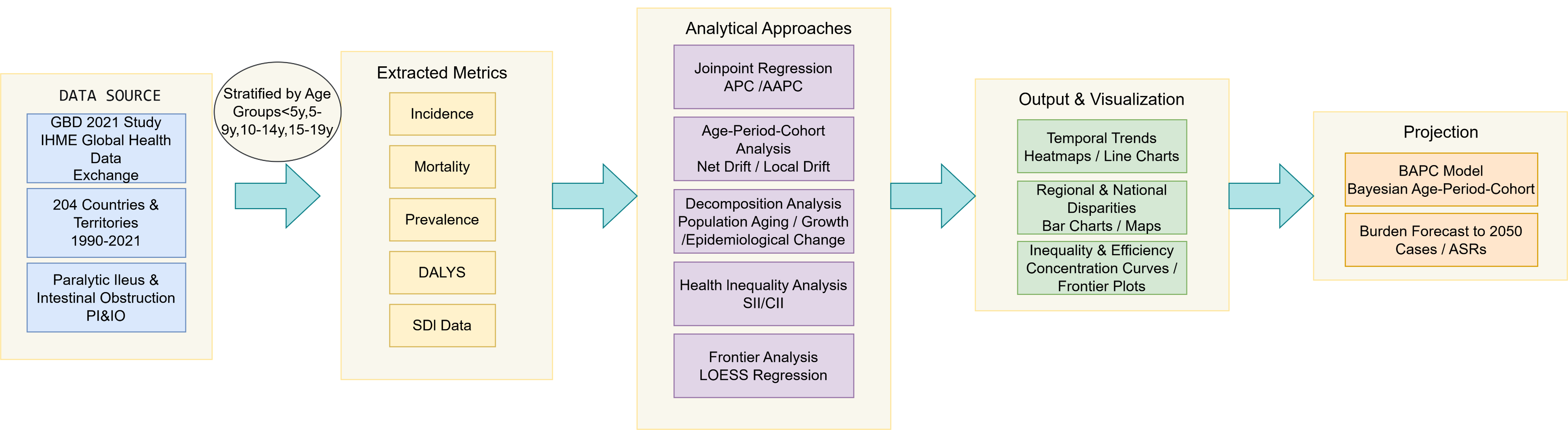

Supplement: Supplementary file 1 [file medi-105-e49668-s001.tif]

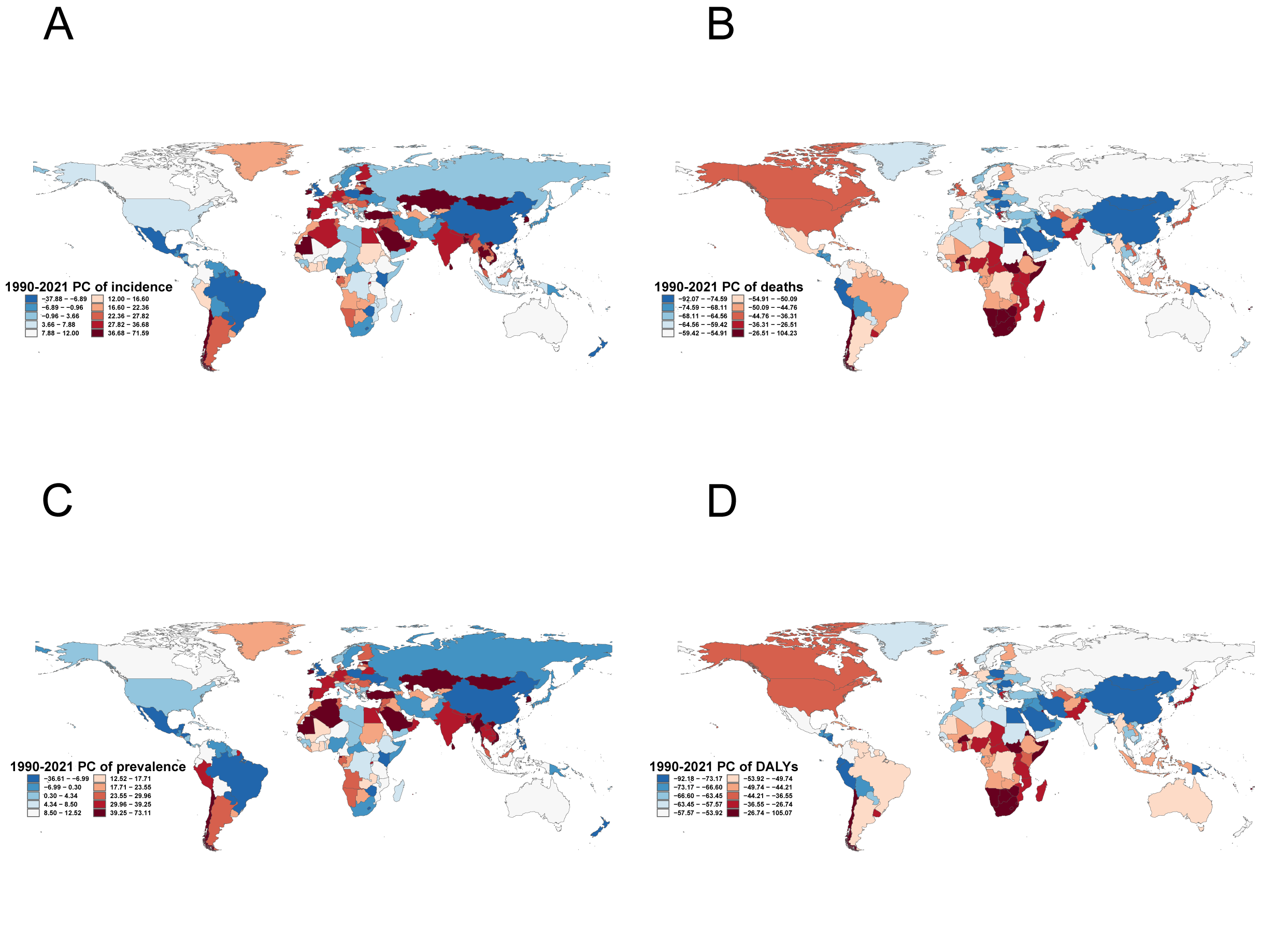

Supplement: Supplementary file 2 [file medi-105-e49668-s002.tif]
